# Evaluation of auditory alerting systems for safe electric scooter operations

**DOI:** 10.1038/s41598-024-80975-1

**Published:** 2025-01-27

**Authors:** Tim Walton, Antonio J. Torija, Richard J. Hughes, Andy S. Elliott

**Affiliations:** Acoustics Research Centre, University of Salford, The Crescent, Manchester, M5 4WT UK

**Keywords:** Engineering, Psychology

## Abstract

It is well understood that a significant shift away from fossil fuel based transportation is necessary to limit the impacts of the climate crisis. Electric micromobility modes, such as electric scooters and electric bikes, have the potential to offer a lower-emission alternative to journeys made with internal combustion engine vehicles, and such modes of transport are becoming increasingly commonplace on our streets. Although offering advantages such as reduced air pollution and greater personal mobility, the widespread approval and uptake of electric micromobility is not without its challenges. Concerns have been raised regarding the safety of such vehicles, most notably related to pedestrian safety of blind and partially sighted individuals, due to the inherently lower sound levels produced by electric vehicles. This study addresses this issue by investigating the use of an Acoustic Vehicle Alerting System (AVAS) for electric scooters by means of a virtual reality experiment and field trials. Eighty-eight participants from four European countries, including thirty-five blind or partially sighted individuals participated across the experiments. Results show high missed detection rates for electric scooter operations without an AVAS in typical city soundscapes (90–97%) and an increase in detectability for all AVAS conditions tested. Modifying AVAS sounds with playback rate and level changes with respect to operational state facilitates detection of deceleration, as well as improving detectability in multiple vehicle scenarios.

## Introduction

Over the last decade, there has been a rapid rise in the number of shared-use, on-demand mobility options available in the world’s urban areas^1,2^. ‘Micromobility’ forms one aspect of this shared-use mobility ecosystem, which is a term used to describe small, lightweight vehicles operated at low speeds that can either be human-powered, such as pedal bicycles, or electric-powered, such as electric bicycles and standing electric scooters^3^. Micromobility offers an alternative solution to ‘first and last mile’ trips and can therefore complement public transportation systems, as well as reducing private car dependence^4,5^. By replacing journeys made with private internal combustion engine vehicles, micromobility modes have the potential to improve quality of life by reducing congestion within our urban environments, whilst lowering emissions and improving air quality^6^. Wider adoption of micromobility solutions could ultimately also lead to a redesign of urban spaces, as car-centric design gives way to a more pedestrian focused approach.

Shared-use standing electric scooters (e-scooters) are one form of micromobility that has seen a prominent growth over the last few years, with an estimated 520,000 shared e-scooters across Europe in 2022, up from 360,000 in 2021^7,8^. These vehicles typically have a maximum speed limit of approximately 20 km/h, a mass not exceeding 35 kg and are designed to carry one person in a standing position with no provision for seating^9^. One potential barrier for widespread acceptance of e-scooters however, both on a subjective and regulatory level, is perceived safety of these vehicles by pedestrians. In a UK Department for Transport survey on perceptions of current and future e-scooter use in the UK, 53% of respondents cited safety issues as one disadvantage of e-scooters^10^ and a comprehensive study on perceptions of an e-scooter trial in Greater Manchester revealed that 45% of respondents had felt unsafe when walking as a result of an e-scooter rider^5^. This figure is increased further still when considering data from respondents with vulnerabilities^5^.

Of particular concern is the safety of blind and partially sighted pedestrians who are within the vicinity of moving e-scooters, as previous research has shown that e-scooters are often undetectable by auditory cues alone when in typical city soundscapes^11^. When considered with data from an Australian study which shows that 40 percent of e-scooters pass within 1 m of at least one pedestrian^12^, it is reasoned that there is a significant risk for e-scooter pedestrian collisions, and indeed this is backed up by a review of e-scooter injury patterns^13^. The safety of blind and partially sighted individuals has been widely considered in the context of electric vehicles (EVs) and hybrid electric vehicles (HEVs)^14–18^ and this has led to a range of regulations that specify minimum noise requirements for quiet running vehicles by means of Acoustic Vehicle Alerting Systems (AVAS)^19–21^. These regulations typically specify minimum AVAS sound levels in one-third octave bands up to a speed of 20–30 km/h, above which sound level measurements show little difference between EVs and internal combustion engine vehicles due to an increased contribution from rolling noise and wind noise^17^. Moreover, requirements specify that AVAS varies proportionally with speed so as to alert pedestrians of acceleration and deceleration. Although AVAS regulations for EVs may include relevant aspects for micromobility modes, differences in vehicle size and weight (and hence different expectations concerning sound character), different use patterns including proximity to pedestrians, and different baseline noise characteristics between the modes, means that it should not be assumed that current AVAS regulations are also appropriate for micromobility.

Whilst research on detectability and annoyance of AVAS for EVs and HEVs is well represented within the literature^22–27^, research on AVAS for e-scooters, and more widely micromobility, is much more limited. Torija et al. presented a small-scale feasibility study using virtual reality (VR) to investigate pedestrian awareness of an approaching e-scooter with and without AVAS. Results indicated that the detection time could be reduced by 0.48 s for an AVAS which increased the e-scooter sound level by 2 dBA at the listener position^28^. Walton et al.^11^ conducted a listening experiment in which acoustic simulations of e-scooter passes were presented via a three-dimensional loudspeaker array, to assess detectability and annoyance of various AVAS sounds in a range of typical city soundscapes. This study highlighted that e-scooters have a low detection rate in typical city soundscapes when using auditory cues alone, and that an additional AVAS can increase detection to a safer distance. It was further shown how the objective metric of ‘Zwicker’s psychoacoustic annoyance’ can help predict AVAS annoyance ratings, whilst ‘partial loudness’ can help predict AVAS detectability when in environmental noise. Expanding on the previous study, Walton et al.^29^ further investigated the relationship between pedestrian auditory detection rates and alert sound level for e-scooters using a controlled, binary-choice listening experiment. A range of e-scooter AVAS conditions and environmental noise levels were presented in the presence of a simplified environmental noise spectrum. Psychometric functions were subsequently derived, resulting in an understanding of auditory detection probabilities as a function of AVAS level, environmental noise level and distance.

These studies are a useful indicator that an e-scooter AVAS may be necessary for auditory detection in city environments, as well as providing some indication of the reproduction level needed depending upon the environment, however, they are limited in that they only consider a simple detection task with participants of one country of residence. The paper presented here offers a significant additional contribution by taking a more *ecologically valid* approach for a more realistic evaluation of e-scooter AVAS under typical urban scenarios. Namely, a range of e-scooter operating conditions are investigated, which have not previously been considered, an acoustically accurate VR simulation method was used and the study was international in nature, with blind and partially sighted participants from across four countries. Furthermore, the ecological validity of this study is enhanced by presenting results from on-street field trials, also conducted with blind and partially sighted participants.

The VR approach that was adopted enabled a perceptually accurate representation of both e-scooter sound and movement, so as to investigate use cases, including passes from behind, speed changes, and multiple e-scooter scenarios, in a controlled and reproducible manner. 360-degree audio-visual recordings of city environments were combined with e-scooter animated graphics and auralisation utilising an acoustic ray-tracing plug-in, which modelled the effects of spherical spreading, ground reflections, air absorption and Doppler shift. For a more accurate representation of the acoustic source directivity, a modification to the plug-in was developed that allowed frequency dependent directivity data, as measured in an anechoic chamber. Experiments were conducted in a range of European cities to gather data from a wide demographic, and with participants who have a range of visual acuity, from blind to sighted. Key features of AVAS sounds have been compared across scenarios, including reproduction level, modification methods with respect to speed, and AVAS character, by means of detectability and annoyance tasks. Whilst contributing to the current understanding of AVAS requirements for micromobility, this study also offers a methodology for evaluating and designing AVAS sounds on a range of transportation modes, in turn informing policy and regulations going forward.

## Methods—VR study

Within the VR experiment, participants were required to complete three detection based tasks and an acceptability rating task including open text responses. In the first task, participants were required to detect an e-scooter passing from behind, with the aim to compare detectability performance of AVAS sounds with continuous features, impulsive features, and combined features. In the second task, participants were required to identify the onset of e-scooter deceleration and also to identify the point at which the e-scooter became stationary after decelerating to a stop. As well as the above AVAS types, AVAS modifiers that reflect operational state were compared, based on audio playback rate modifications that alter both the frequency and modulation rate of the AVAS sound with respect to speed. The third task investigated detectability performance for multiple e-scooter scenarios and participants were required to detect an e-scooter passing from behind when two other e-scooters were operational within the scene. The objective of this task was to compare the AVAS sound types and also to investigate if a speed dependent AVAS can aid in detectability performance for multiple e-scooter scenarios. Finally, a rating task was conducted whereby participants gave acceptability ratings and made further comments on the presented AVAS sounds. Further details are presented in the sections below.

### Participants

$N =$ 63 participants were recruited for the study across a range of locations; $N =$ 26 participated in Manchester (UK), $N =$ 5 in London (UK), $N =$10 in Stockholm (Sweden), $N =$ 9 in Milan (Italy) and $N =$13 in Madrid (Spain). Participants were recruited through an internal participant database and also through project stakeholders, who included the following blind organisations from across Europe: The Royal National Institute of Blind People (RNIB), Synskadades Riksförbund (SRF), Unione Italiana dei Ciechi e degli Ipovedenti (UICI) and Organización Nacional de Ciegos Españoles (ONCE). $N =$ 42 participants were male (67%) and $N =$ 21 female (33%) and age data was recorded in ranges, with the youngest participants falling within the age range of 18-25 and the oldest within the range ‘66 or older’. $N =$ 31 participants identified as blind or partially-sighted (49%) with the remaining $N =$ 32 participants (51%) having normal or corrected-to-normal vision. $N =$ 3 participants self-reported slight hearing loss in one ear (5%), however due to the mildness of the reported conditions, their participation was deemed appropriate (pending participant reliability analysis). The remaining $N =$ 60 participants (95%) self-reported normal hearing.

### Apparatus

A Vive Pro 2 (HTC Corporation) head-mounted display (HMD) was used to present the experiment, with a resolution of 2448 $\times$ 2448 pixels per eye and a 120$^{\circ }$ horizontal field of view. A Vive VR controller was used to submit responses during the detection-based tasks. Audio was presented with Sennheiser HD 650 open-back headphones, with an RME ADI-2 digital to analogue converter and headphone amplifier. Calibration of reproduced audio levels was undertaken using a B&K Type 4128-C Head and Torso Simulator, a Norsonic 336 microphone amplifier, a BSWA 308 sound level meter (class 1), and a B&K 4230 sound level calibrator. The 360 degree audio-visual recordings were made using an Insta360 Pro 2 camera (8K resolution rendered to 5K), a Soundfield ST450 Ambisonic microphone, and a Zoom F8n Field Recorder. A B&K Type 2250 sound level meter (class 1) was used to log environmental sound pressure levels during the 360 degree audio-visual recordings for calibration purposes.

### Simulation

**Fig. 1:**
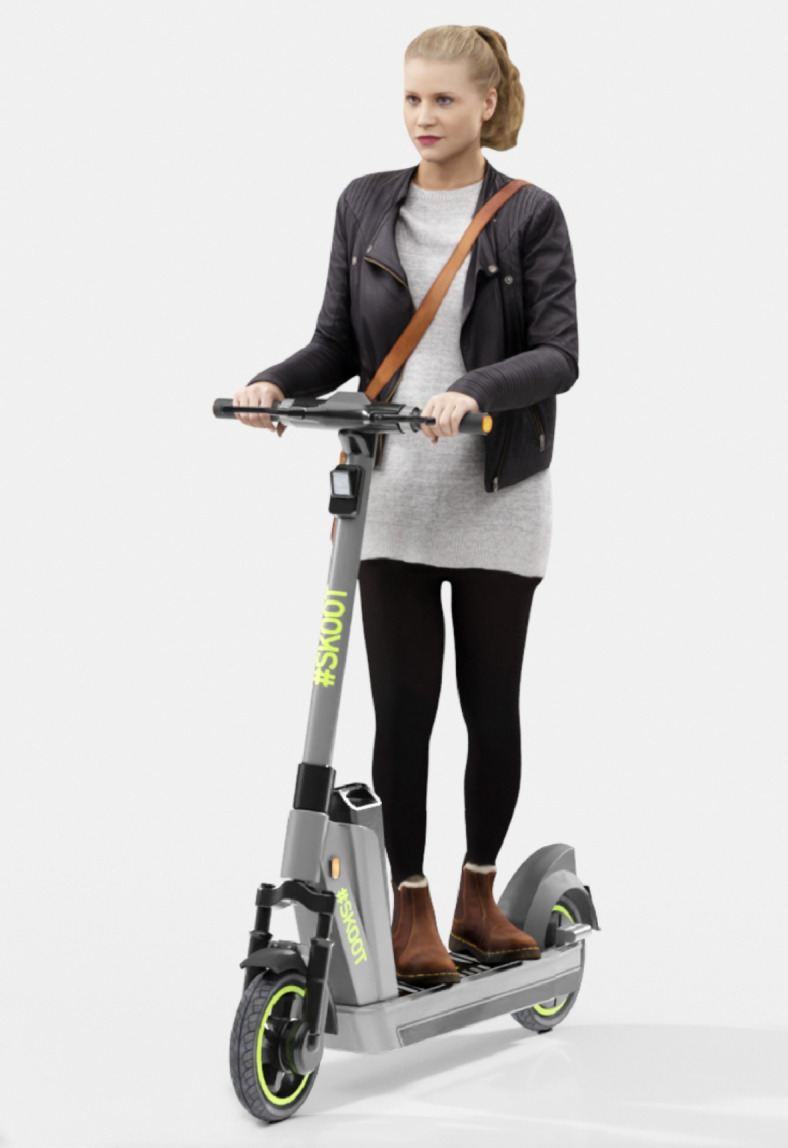
Example of the computer generated e-scooter graphics used in the VR experiment. The models were generated with photogrammetry and included animation of both rider and e-scooter from motion capture data.

The VR environment was built via the Unity game engine (2022.1.13f1, Unity Technologies^30^) and combined 360 degree audio-visual recordings with simulations of e-scooter passes. The e-scooter graphics were generated with the 3D computer graphics software Blender (Blender Foundation^31^) using photogrammetry and included animation of both rider and e-scooter from motion capture data^32^, see Fig. 1.

Auralisations comprised dynamic binaural reproduction via the Unity engine using the built-in default head related transfer function database. Additional spatial audio features were simulated through use of the plugin Steam Audio (version 4.1.2). Environmental scene sounds, captured as first-order Ambisonic audio recordings with the Soundfield ST450 Ambisonic microphone, were decoded using the Steam Audio Ambisonic Decoder. E-scooter sounds, including ground reflections based on a concrete surface, spherical spreading, air absorption, and Doppler shift, were rendered using the Steam Audio Spatializer and custom source directivity scripts.

Steam Audio allows for simulation of first-order frequency independent source directivity, which improves on Unity’s built-in simple omnidirectional audio source type. However, this only allows relatively simple control, and more realistic sound sources such as an e-scooter AVAS including loudspeaker and housing, will have a frequency dependent response with more complex directional behaviour. To further aid auralisation authenticity, therefore, a more accurate implementation of source directivity was developed. Anechoic measurements with horizontal receiver angle were taken of an e-scooter with a representative loudspeaker driver (28 mm diameter) attached to the stem. The data was analysed in MATLAB (R2021a, MathWorks) in a set of audio bands, normalised to the on-axis (front of scooter) response, and encoded to a set of frequency dependent real spherical harmonic (SH) coefficients. Unity scripts were created to generate an audio source master to control a series of auto-generated children (audio bands), and a SH calculator (for a given source-receiver pair). Each child contained scripts for audio band filtering and directivity SH decoding (using loaded MATLAB generated filter and SH coefficients) along with the necessary audio components. E-scooter sounds were simulated in octave bands from 250 Hz to 8.0 kHz and at SH order $N =$ 8.

### Stimuli

#### Environments

360 degree audio-visual recordings were obtained from three locations which represent typical use cases for e-scooter operation; a shared use path in a city park (ENV1), a shared use city concourse (ENV2) and a busy city road with a bus/bicycle lane (ENV3). As well as representing typical use cases, these environments offered varied visual and acoustic properties, with further details presented in Table 1. Sound pressure level measurements were taken during the audio-visual recordings and these were used to calibrate the scenes to representative levels. More details of these environments can be found within the supplementary information provided.

#### E-scooter baseline

To enable acoustically accurate simulation of e-scooter movements, baseline e-scooter noise was determined through acoustic measurement. Sound level measurements were taken of typical e-scooter passes on asphalt at 1 m distance, with a resulting broadband sound pressure level of 58 dB $L_{\textrm{AFmax}}$ (equivalent to 52 dB $L_{\textrm{AFmax}}$ at 2 m distance when assuming spherical propagation from a point source), where $L_{\textrm{AFmax}}$ refers to the maximum sound level with ’A’ frequency weighting and fast time weighting during the measurement period. Furthermore, on-scooter audio was recorded whilst the e-scooter was travelling at 20 km/h, with the microphone positioned approximately 1 m from the edge of the scooter and 1.7 m from the ground. This represents the position of a pedestrian and includes any contribution from tyre noise, wind noise and motor noise. This audio was then processed with Unity to simulate baseline e-scooter movements and additional AVAS sounds were added as described in the following section. Further details on the e-scooter baseline have been presented in previous literature^11^.

#### AVAS

An effective AVAS sound should account for human auditory frequency sensitivity^33^, which peaks approximately between 1 kHz and 5 kHz, should include components lower than 1 kHz to accommodate for individuals with high frequency hearing loss^33^, and should avoid components below 200 Hz so as to limit unwanted noise propagation over long distances and intrusion through typical building facades^34^. Moreover, AVAS sounds with few harmonics and prominent amplitude modulation have been shown to optimise detectability^22^, and previous studies have shown that tones based around sine waves best optimise detectability and annoyance, with promising results from both continuous and impulsive type sounds^11^.

Three AVAS sounds (AVAS_type_) at two reproduction levels each (AVAS_level_), were investigated during this study, as informed by the above research and considerations of typical e-scooter hardware capabilities, namely considerations of reproduction frequency ranges of small-diaphragm loudspeaker drivers, which typically offer poor low frequency performance. The three AVAS sounds were based around the chromatic notes of G4 (392 Hz), D5 (587 Hz), G5 (784 Hz) and D6 (1175 Hz), which when processed with a 1% per km/h playback rate increase, resulted in frequencies of 478 Hz, 716 Hz, 957 Hz and 1434 Hz at 20 km/h. This range of frequencies was selected as it satisfies the above considerations when the e-scooter is at a typical operational speed. As it was shown previously that both continuous and impulsive type sounds may offer good detectability^11^, the three AVAS sounds were selected so as to compare the performance of continuous type sounds, impulsive type sounds, and sounds that contain a mixture of both continuous and impulsive components. In this paper, continuous sounds refer to those which are perceived as a ‘chord’ and have no perceivable ‘attack’, whereas impulsive sounds are characterised by a series of short, repeated impulses with a rapid attack and delay. All sounds were created within software synthesisers within a digital audio workstation and further modified to reflect operational speed in Python by altering playback rate and output level. It should be noted that the AVAS sounds used in this study were the same as those used by Walton et al.^29^

**Fig. 2:**
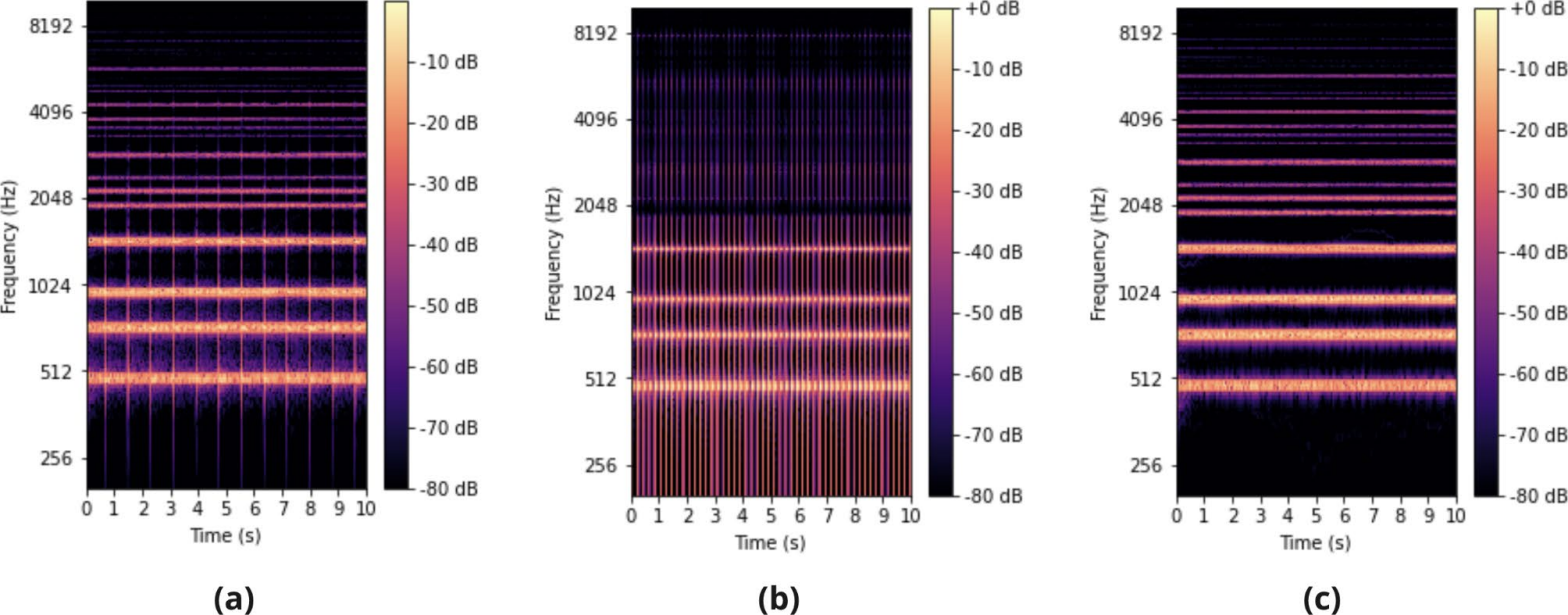
Spectrograms for AVAS stimuli S_cont_ (**a**), S_imp_ (**b**) and S_mix_ (**c**), at 20 km/h. Colour represents relative amplitude in decibels, y-axis represents frequency (Hz), and x-axis represents time (s).

With regards to reproduction levels, 56 dB $L_{\textrm{AFmax}}$ (2 m distance) was used as this corresponds to the minimum requirements specified in UNECE Regulation 138 for quiet running vehicles^19^, as well as 66 dB $L_{\textrm{AFmax}}$ (2 m distance), which corresponds to a + 10 dB uplift on UNECE requirements. Spectrogtrams of the AVAS sounds are shown in Fig. 2 with more details outlined in Table 1. In summary, AVAS sounds of a continuous nature, an impulsive nature, and a combination of continuous and impulsive components were compared for two reproduction levels across a range of tasks. Furthermore, the AVAS sounds were processed through an audio playback rate and level change algorithm to reflect operational speed, as outlined in the following section.

**Table 1:** Audio stimuli details with calibration levels.

Stimuli Ref	Description	Calibration level
ENV1	City park environment, characterised by distant road traffic noise	49 dB $L_{\textrm{Aeq}}$
ENV2	Shared use concourse, characterised by plant and machinery noise	55 dB $L_{\textrm{Aeq}}$
ENV3	Busy city road, characterised by dominant road traffic noise	70 dB $L_{\textrm{Aeq}}$
S_base_	Baseline e-scooter audio recording	52 dB $L_{\textrm{AFmax,}}$ 2 m
S_cont_	Continuous AVAS sound based around frequency components of 478 Hz, 728 Hz, 956 Hz and 1433 Hz, and an amplitude modulation rate of 4.9 Hz, when at 20 km/h	56/66 dB $L_{\textrm{AFmax,}}$ 2 m
S_imp_	Impulsive AVAS sound based around frequency components of 478 Hz, 716 Hz, 957 Hz and 1434 Hz, and an impulse rate of 4.9 Hz, when at 20 km/h	56/66 dB $L_{\textrm{AFmax,}}$ 2 m
S_mix_	Combined continuous and impulsive AVAS sound using S_cont_ and an impulsive element, with the same frequency components as above, and an impulse rate of 1.15 Hz when at 20 km/h. A lower impulse rate was chosen to enable better perception of the continuous element	56/66 dB $L_{\textrm{AFmax,}}$ 2 m

For AVAS stimuli, two levels were compared (56 dBA and 66 dBA).

### Procedure and design

Prior to conducting the subjective experiment, the following procedure and design was approved by the Ethics Committee of the University of Salford, UK (application ID 8147). All methods were carried out in accordance with relevant guidelines and regulations.

#### Consent and demographics

The experimental procedure was fully explained to participants prior to commencement and informed consent was obtained. All written material was professionally translated from English to Spanish, Italian and Swedish to correspond to country of participation. Furthermore, all written material was available as an audio description for blind and partially sighted individuals. Participants completed a demographic survey to record age, gender, e-scooter usage, hearing impairments and visual acuity.

#### Task 1—detection

Following HMD fitting and calibration adjustments with the built-in interpupillary distance (IPD) dial, participants were presented with a simple detection task within the virtual environment. For each trial, a 30–40 s segment of one of the audio-visual environments was presented. After a random delay of between 4 and 8 seconds, an e-scooter was simulated to travel from 60 m behind the participant, to 60 m in front of the participant, with a closest approach of 2 m, either to the left or the right depending on the scene. Participants were instructed to press the trigger on the controller as soon as they heard an e-scooter approaching. Furthermore, they were instructed only to explore the front hemisphere within the VR environment, so as not to try and locate the e-scooter with visual cues. The task consisted of 27 trials: 18 containing S_base_ and AVAS (all combinations of three environments, three AVAS types, and two levels), 3 containing S_base_ only (three environment types), and 6 containing no e-scooter simulation (two in each of the three environments). Trials where there was no e-scooter movement were included so as to make the task less predictable. All trials were randomised and no scene was presented twice in a row. Please refer to the supplementary information for an overview of presented variable combinations alongside an example video of a trial from this task. A short training session was provided before the main detection task containing ENV1 and the AVAS sounds at the 66 dB $L_{\textrm{AFmax}}$ level. This was to ensure the participants were comfortable with the task and to familiarise them with the e-scooter AVAS sounds.

#### Task 2—deceleration

Vehicles travelling in parallel and decelerating has previously been identified as a safety critical scenario for blind pedestrians in the context of quiet running vehicles^15,16^. Such a scenario occurs when a vehicle is slowing to turn into a side-road, potentially travelling across the path of the pedestrian. Moreover, identification of deceleration and stopping is necessary for blind pedestrians to safely navigate pedestrian crossings. To convey speed information, current UNECE AVAS regulations specify that the AVAS varies proportionally with speed by an average of at least 0.8% per 1 km/h in the speed range from 5 to 20 km/h inclusive when driving in a forward direction^19^. Previous research regarding identification of vehicle operating conditions through auditory cues has highlighted that both level changes and frequency changes can lead to an easier detection of operating state^35^, and that sound fluctuations mirroring speed variations can enhance detectability^36,37^.

To investigate the perception of e-scooter operating states, a deceleration task was conducted with four AVAS modifiers (AVAS_mod_): playback rate increase of 1% per km/h (PR1), playback rate increase of 2% per km/h (PR2), playback rate increase of 1% per km/h plus level change (PR1,L) and playback rate increase of 2% per km/h plus level change (PR2,L). The playback rate algorithm resamples the audio file and therefore influences both frequency and amplitude modulation rate. This technique was chosen as it represents a more computationally simple procedure in comparison to time stretching with static pitch, or pitch shifting with static playback rate, which both require signal decomposition into analysis frames^38^. This is an important consideration for implementation of micromobility AVAS, where it is desirable that unit cost is minimised where possible. The level change condition reduced the AVAS gain by 0.6 dB per km/h for speeds between 10 and 20 km/h and 2 dB per km/h for speeds between 1 and 10 km/h. Below 1 km/h the AVAS was disabled, and above 20 km/h the AVAS was at maximum volume. This level change was chosen to comply with UNECE Regulation 138, which specifies a reduction in minimum AVAS level of 6 dB between 20 and 10 km/h^19^. Both playback rate and level change modifications were applied to the AVAS sounds by means of a Python script.

During the task, the e-scooter either passed 2 m to the side without slowing, or decelerated from 20 to 0 km/h over the course of 7.5 m, taking 2.7 s, coming to a stop 1 m behind the participant. This stopping range corresponds approximately to the minimum technical specifications outlined for e-scooters by the UK Government, who specify a minimum stopping distance of 7 m from a speed of 15.5 mph^39^. Participants were required to press the trigger when they first detected the e-scooter to be slowing and again when they detected that the e-scooter had come to a stop. There were 15 trials in total, all within ENV1, as the low environmental noise level of this scene ensured that the AVAS sounds and respective modifiers were audible. 12 stopping operations were presented (all combinations of 3 AVAS types at the 66 dBA level and 4 AVAS modifiers), and 3 pass-bys (3 unmodified AVAS types). The order of all trials was randomised. Please refer to the supplementary information for an overview of presented variable combinations alongside an example video of a trial from this task.

#### Task 3—multiple source

**Fig. 3:**
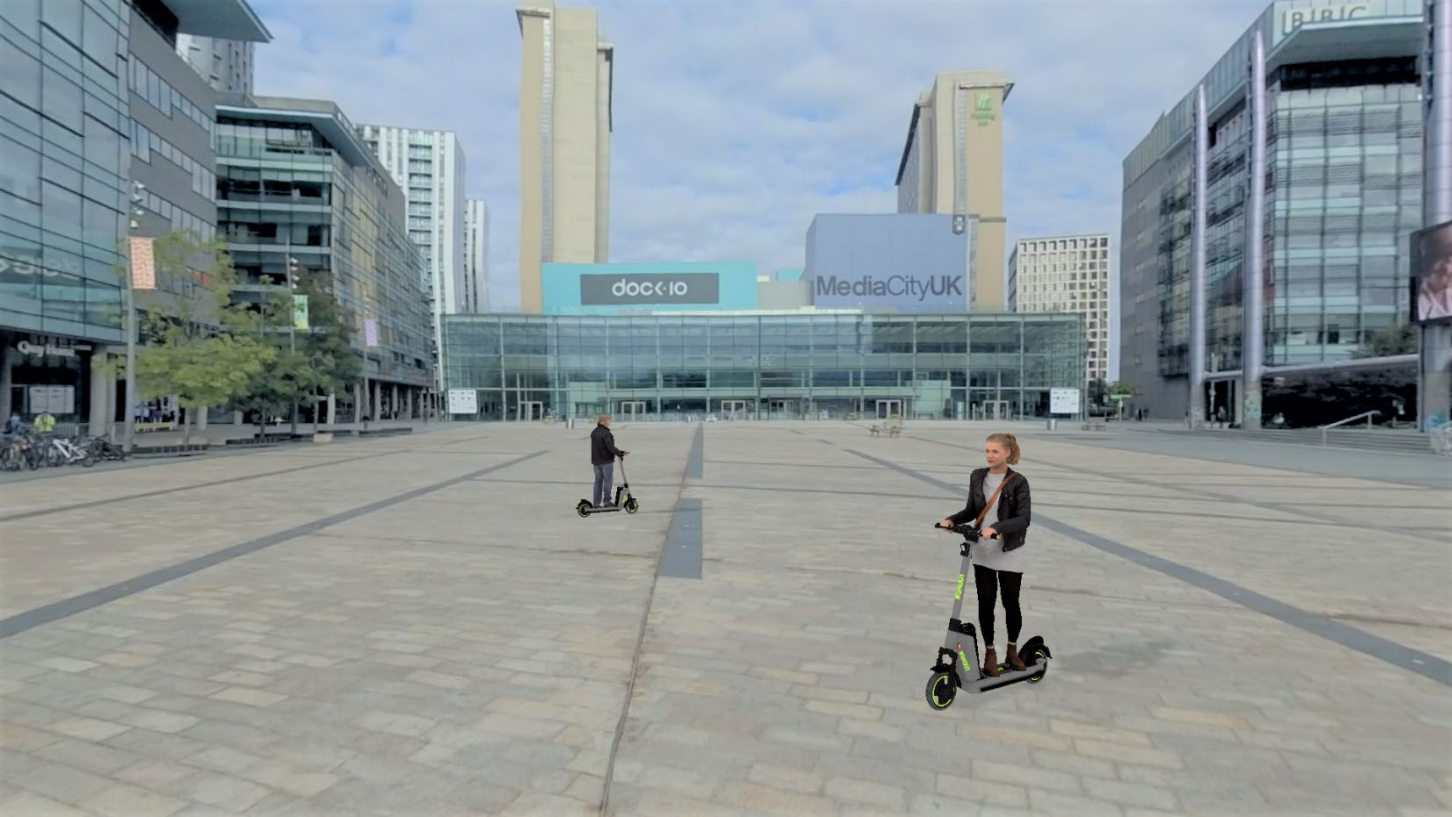
Screenshot from the multiple source task showing a spatial crop of the 360 degree video environment with computer generated e-scooter graphics. Within this task, two e-scooters were riding in a circular trajectory in front of the participant (centre point of 10 m from the participant, radius of 5 m) and a third e-scooter passed from behind. Participants were required to detect the approaching e-scooter from behind.

The majority of studies on AVAS to date have primarily focused on detection and annoyance of single vehicles. However, an important consideration with regards to both pedestrian safety and soundscape design is how AVAS sounds from multiple vehicles interact. Discussions on how concurrent AVAS sounds may combine to produce disharmonious urban soundscapes have previously been considered^40–42^, but studies looking at the interaction of multiple AVAS sounds on detectability are lacking. A multiple source detection task was considered in this study, in which three e-scooters were operational within the scene. During this task, two e-scooters were riding in a circular trajectory in front of the participant (centre point of 10 m from the participant, radius of 5 m), as seen in Fig. 3, and a third e-scooter passed from behind with parameters as in the previous tasks. Participants were required to identify when they heard the third scooter approaching from behind, in the presence of the distraction AVAS sounds. There were 12 trials in total, all within ENV2; for each of the three AVAS types at the 66 dBA level, there was a trial both with and without a pass from behind, with all three e-scooters using the same AVAS sound (referred to as the AVAS_speed_ ‘speed independent’ condition). Additionally, trials were conducted with the same variable combinations, but in which the frontal e-scooters had a modified AVAS (playback rate increase of 1% per km/h) to reflect a speed of 13 km/h, to simulate the scenario where multiple e-scooters are operational with different velocities (referred to as the AVAS_speed_ ‘speed dependent’ condition). The order of all trials was randomised. It should be noted that the environmental noise level of ENV2 was reduced to 51 dB $L_{\textrm{Aeq}}$ in this task, to increase the relative contribution from the distraction AVAS sounds. Please refer to the supplementary information for an overview of presented variable combinations alongside an example video of a trial from this task.

#### Task 4—acceptability rating

Following the VR detection tasks, an audio-only acceptability rating task was completed using a single page, multiple-stimulus graphical user interface developed with MAX/MSP (Cycling ’74). E-scooter passes in ENV1 with each of the 66 dBA AVAS sounds were exported from Unity and randomly assigned to buttons *“A”*, *“B”* and *“C”*, and participants were required to rate each sound with regards to acceptability, prompted by the following questions: *“Based on your experiences in VR, please rate each sound in terms of how acceptable it is for use as an e-scooter alert sound”*. Ratings were given on a 5-point scale with verbal anchors of *“not at all acceptable”*, *“slightly acceptable”*, *“moderately acceptable”*, *“very acceptable”* and *“completely acceptable”*. A text box was also provided with the following prompt: *“please specify which is your favourite sound and enter any other comments about the sounds you wish to discuss”*. Audio description capabilities enabled navigation and input by blind and partially sighted participants.

#### Data processing and statistical analysis

The collected data were statistically analyzed using the software package SPSS Statistics v.28 (IBM Corp.). A threshold level of 5% (*p* = 0.05) was used for statistical significance throughout the analysis.

## Results—VR study

### Task 1—detection

#### Participant screening

Prior to further analysis, participant reliability was checked by comparing the number of false detections across participants, to evaluate comprehension of the task. False detections were defined as responses made during trials with no pass, or for trials where a pass occurred, detections made prior to the e-scooter pass commencing. The false detection rate from $N =$ 1 participants was identified as an outlier when compared to the sample as a whole (greater than 1.5 times the interquartile range above the upper quartile), based on a median of 1 and an upper adjacent of 4. As such, this participant was excluded from the following analysis of the task.

#### Missed detections

**Fig. 4:**
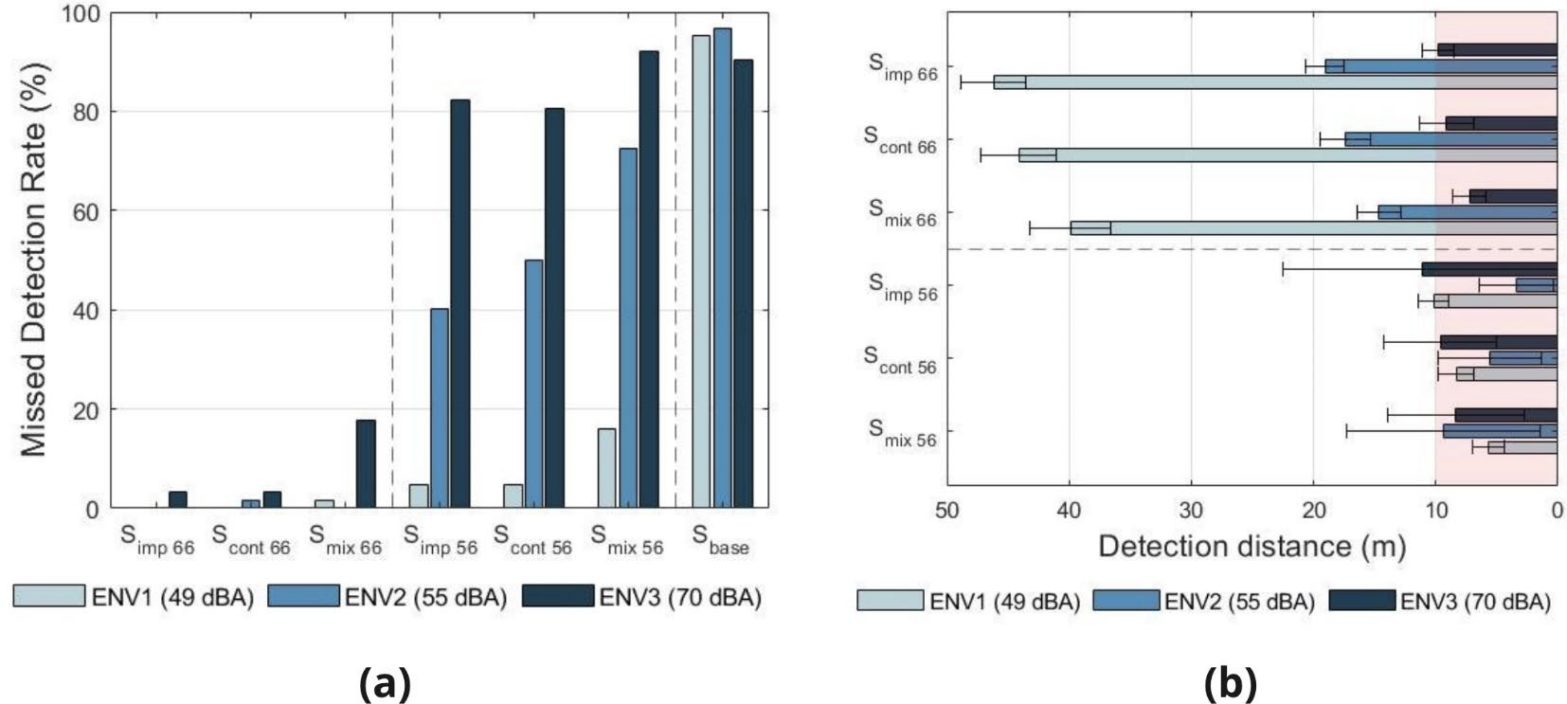
Task 1 results. (**a**) Missed detection rate by AVAS and environment. (**b**) Mean detection distance by AVAS and environment. Red shading indicates ‘risk’ area. Error bars show 95% confidence intervals.

Missed detections were defined as trials where an e-scooter pass occurred, but either no response was made, or the response was made after the e-scooter had passed the participant (reaction distance RD < 0). Figure 4 presents the missed detection rate by AVAS_type_, AVAS_level_ and ENV variables. To examine the statistical significance of the missed detection rates, chi-square tests of independence were performed. A significant relationship was observed between missed detection rate and ENV ($\chi ^{2}$ = 117.84, df = 2, $p < 0.001$), AVAS_level_ ($\chi ^{2}$ = 579.03, df = 2, $p < 0.001$), and AVAS_type_ ($\chi ^{2}$ = 15.09, df = 2, $p < 0.001$). To further examine the relationship between AVAS_type_ and missed detection rate, pairwise comparisons including Bonferroni corrections were conducted. The number of missed detections associated with S_mix_ was significantly different ($p < 0.05$) to those associated with S_imp_ and S_cont_, however, no significant difference was observed between S_imp_ and S_cont_. From these results, it can be concluded that without an additional AVAS (S_base_), the e-scooter was typically inaudible prior to passing the participant in all environments, with missed detection rates between 90% and 97%. With the addition of a 66 dBA AVAS, missed detection rates were typically 3% or less, with the exception of S_mix_ within ENV3 (18%). In the case of a 56 dBA AVAS, missed detections varied more prominently with environment, with means of 9% for ENV1, 54% for ENV2 and 85% for ENV3. AVAS condition S_mix_ resulted in significantly more missed detections in comparison to S_cont_ and S_imp_.

#### Detection distance

Figure 4 presents the mean detection distance by AVAS and environment. Within the following results, a positive detection distance refers to detection made prior to the e-scooter passing the participant. A ‘risk’ area is defined as RD < 10 m, based on the minimum stopping distances required for e-scooters on UK roads^39^, in addition to knowledge of braking response times for e-scooters^43^. Within this risk area, e-scooter riders may not have sufficient time to prevent a collision if a pedestrian steps into the path of the e-scooter. It should be noted that responses made after the e-scooter had passed the participant have been excluded from the analysis, to enable meaningful comparison between blind and sighted participants. In order to evaluate the significance of detectability differences between AVAS conditions, a linear mixed model analysis was conducted on responses associated with the additional AVAS sounds (i.e. excluding S_base_). As fixed effects in the model, the main effects of AVAS_type_ (S_imp_, S_cont_, S_mix_), AVAS_level_ (56 dBA, 66 dBA), ENV (ENV1, ENV2, ENV3), age group and visual acuity group were used. The two-way interaction between ENV and AVAS_level_ was also included in the model. To account for differences between individuals, variable Participant was used as a random effect in the model, including intercepts. Visual inspection of residual plots did not reveal any deviations from homoscedasticity or normality. The model reveals that the main effects of AVAS_type_ (*F*(2,749.09) = 18.80, $p < 0.001$), AVAS_level_ (*F*(1,761.77) = 621.42, $p < 0.001$), ENV (*F*(2,756.48) = 384.33, $p < 0.001$) and the interaction between ENV and AVAS_level_ (*F*(2,756.25) = 256.33, $p < 0.001$) all significantly influenced detection distance. The main effects of visual acuity group (*F*(1,51.09) = 2.04, *p* = 0.159) and age group (*F*(5,52.48) = 1.40, *p* = 0.241) did not significantly influence detection distance. To investigate the main effect of AVAS_type_ further, post hoc pairwise comparisons were made with Bonferroni corrections applied. The detection distances of AVAS_type_ S_mix_ were significantly different to those of S_cont_ (2.751, $p < 0.001$) and S_imp_ (3.949, $p < 0.001$), however the detection distances of S_cont_ and S_imp_ showed no significant difference (1.197, *p* = 0.169).

These results indicate that the 66 dBA AVAS conditions provided sufficient auditory detectability in ENV1 and ENV2, however for ENV3, detection distance was below the identified risk threshold. For the 56 dBA AVAS conditions, large variance in the data is seen for ENV2 and ENV3, as high missed detection rates reduced the number of data points. For ENV1, AVAS condition S_imp_ has an associated detection distance approximately equal to the risk threshold, with detection distances for the other AVAS conditions below the risk threshold. The statistical analysis shows that AVAS condition S_mix_ has a significantly shorter detection distance in comparison to S_cont_ and S_imp_ overall.

### Task 2—deceleration

#### Participant screening

In the case of the deceleration task, false detections were defined as responses made when either no deceleration occurs, or when the response is given before the deceleration onset time. The false detection rates from $N =$ 4 participants were identified as outliers when compared to the sample as a whole, based on a median of 0 and an upper adjacent of 2. As such, these participants were excluded from the following analysis of this task.

#### Analysis of reaction times

**Fig. 5:**
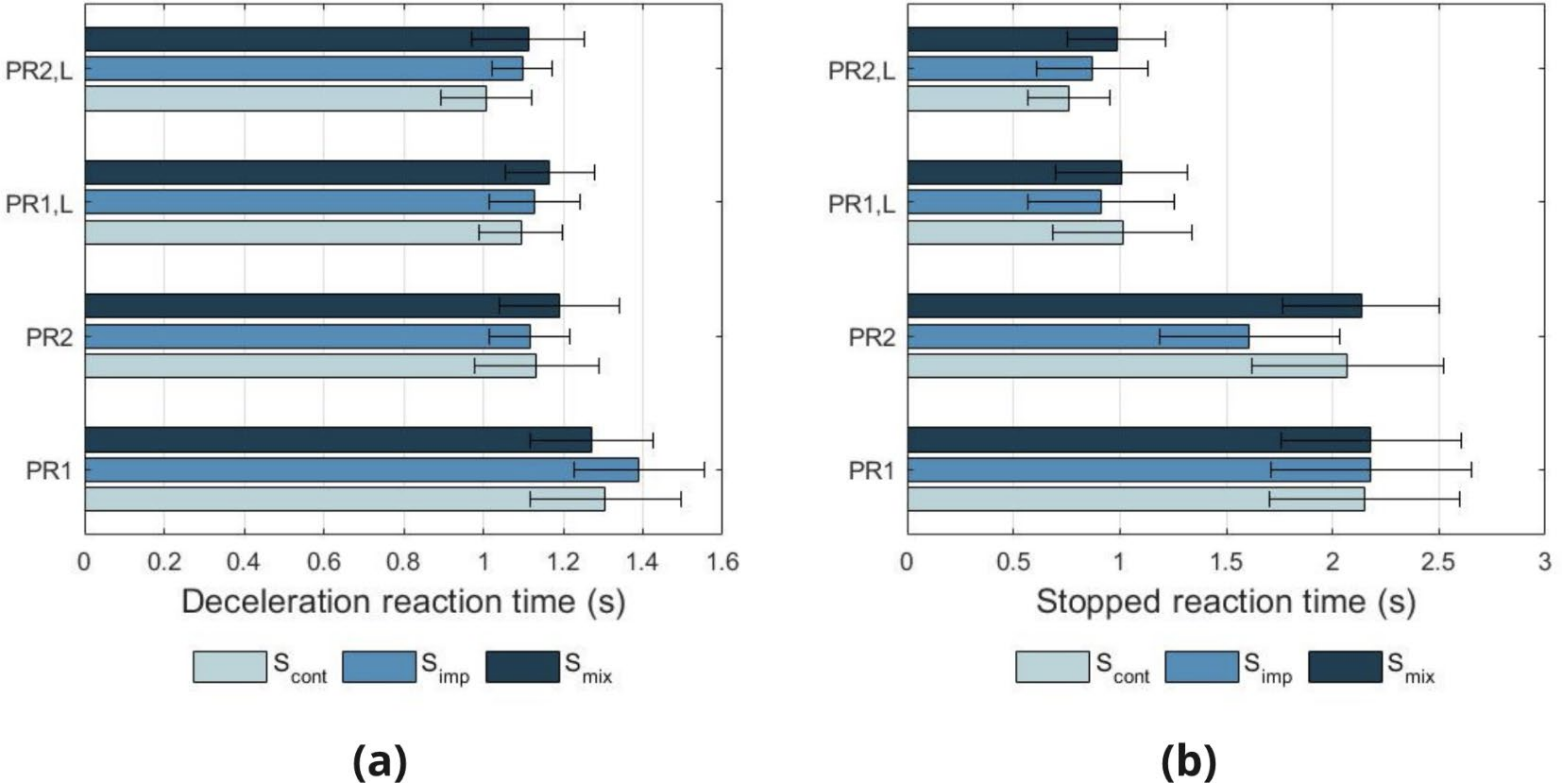
Reaction times after onset of deceleration by AVAS_type_ and AVAS_mod_ (**a**) and reaction times after e-scooter had become stationary by AVAS_type_ and AVAS_mod_ (**b**).

During the deceleration task, participants were required to make a response when they detected that the e-scooter had started decelerating, and also when they detected that the e-scooter had become stationary. Figure 5 presents the reaction times for detection of deceleration and stopping with respect to AVAS_type_ and AVAS_mod_. In order to evaluate the significance of reaction time differences between conditions, a linear mixed model analysis was conducted on responses. As fixed effects in the model, the main effects of AVAS_type_ (S_imp_, S_cont_, S_mix_), AVAS_mod_ (PR1, PR2, PR1,L, PR2,L), age group and visual acuity group were used. To account for differences between individuals, variable Participant was used as a random effect in the model, including intercepts. Visual inspection of residual plots did not reveal any deviations from homoscedasticity or normality.

The model reveals that the main effect of AVAS_mod_ has a significant influence on deceleration reaction time (*F*(3,470.08) = 12.68, $p < 0.001$), however non-significant effects were observed for AVAS_type_ (*F*(2,466.69) = 2.03, *p* = 0.132), age group (*F*(5,49.69) = 2.37, *p* = 0.052) and visual acuity group (*F*(1,49.79) = 0.60, *p* = 0.444). Post hoc analysis with Bonferroni adjustments revealed small but significant differences in reaction times between AVAS_mod_ conditions PR1 and PR2 (0.180, $p < 0.001$) and conditions PR1 and PR2,L (0.182, $p < 0.001$). With stopped reaction time as the dependent variable, the model reveals that the main effect of AVAS_mod_ is significant (*F*(3,473.25) = 67.09, $p < 0.001$), as well as AVAS_type_ (*F*(2,465.68) = 3.20, *p* = 0.042). Non-significant effects were observed for age group (*F*(5,48.62) = 1.16, *p* = 0.342) and visual acuity group (*F*(1,48.78) = 1.66, *p* = 0.203). Post hoc analysis with Bonferroni adjustments revealed significant differences in reaction times between AVAS_mod_ conditions PR1 and PR1,L (1.358, $p < 0.001$), PR1 and PR2,L (1.456, $p < 0.001$), PR2 and PR1,L (1.075, $p < 0.001$), and PR2 and PR2,L (1.173, $p < 0.001$).

These results show that for the detection of deceleration, a playback rate increase of 2% per km/h including level change, resulted in the fastest reaction times, however differences between all conditions were small. When making detections on when the e-scooter had become stationary, a clear distinction is seen between AVAS_mod_ conditions with a level change, to those without. This result suggests that participants could more easily identify when an e-scooter had come to a standstill for conditions when the AVAS sound ceases at zero velocity, in comparison to an AVAS which continues to be audible at zero velocity. With regards to comparisons between a 1% and 2% playback rate increase, minimal differences are seen within the responses.

### Task 3—multiple source

#### Participant screening

In the case of the multiple source task, false detections were defined as responses made during trials with no e-scooter pass from behind, or for trials where a pass occurred, detections made prior to the e-scooter pass commencing. Four participants were identified as outliers when compared to the sample as a whole, based on a median of 0 and an upper adjacent of 2. As such, these participants were excluded from the following analysis of the task.

#### Missed detections

**Fig. 6:**
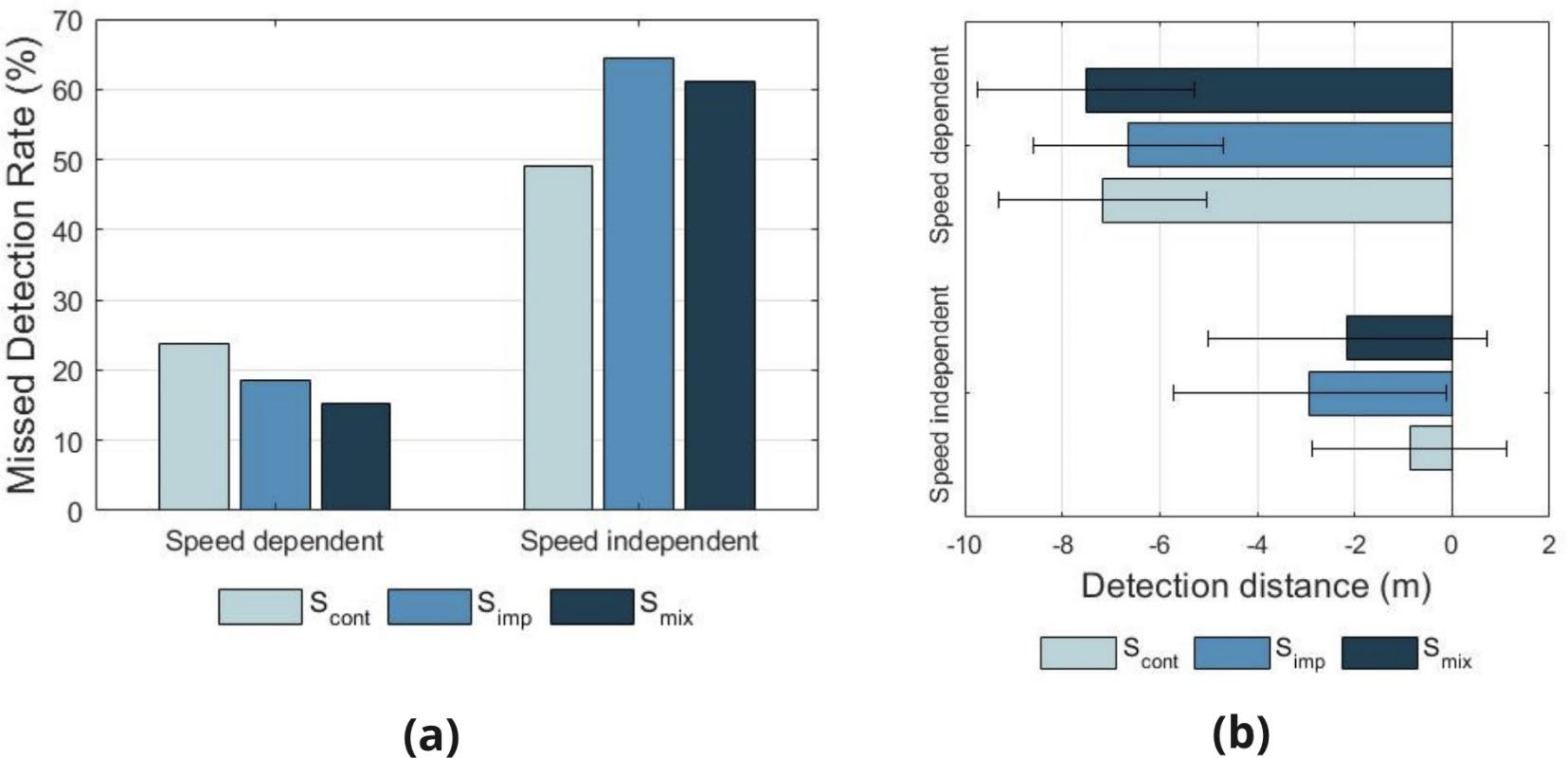
Missed detection rates (**a**) and mean detection distances (**b**) by AVAS_type_ and additional e-scooter AVAS_speed_ group for the multiple source task.

As with Task 1, missed detections were defined as trials where an e-scooter pass occurred, but either no response was made, or the response was made after the e-scooter had passed the participant. Figure 6 presents the missed detection rate for Task 3, by AVAS.

Chi-square tests of independence reveal a significant difference in missed detection rates between the two additional e-scooter AVAS_speed_ conditions (speed independent / speed dependent) ($\chi ^{2}$ = 56.69, df = 1, $p < 0.001$).

#### Analysis of detection distance

Figure 6 presents the mean detection distances for Task 3, by AVAS condition. To evaluate the significance of the differences seen in detection distance between conditions, a linear mixed model analysis was conducted on responses. As fixed effects in the model, AVAS_type_ (S_imp_, S_cont_, S_mix_), AVAS_speed_ (speed independent, speed dependent), age group and visual acuity group were used. To account for differences between individuals, variable Participant was used as a random effect in the model, including intercepts. Visual inspection of residual plots did not reveal any deviations from homoscedasticity or normality. The model reveals that the only variable with a significant effect on detection distance is AVAS_speed_ ($F(1,271.39) = 34.40$, $p < 0.001$), whilst AVAS_type_ ($F(2,267.30) = 0.38$, $p = 0.686$), age group ($F(5,51.00) = 0.29$, $p = 0.918$) and visual acuity group ($F(1,50.34) = 0.13$, $p = 0.717$) show non-significant effects.

These results suggest that participants could not easily identify a passing e-scooter in a multiple e-scooter scenario for speed independent AVAS conditions, that is, when all e-scooters are producing the same sound. By including a modified AVAS to reflect differences in speeds of the multiple e-scooters, detection rates and distances were significantly improved.

### Task 4—acceptability rating

**Fig. 7:**
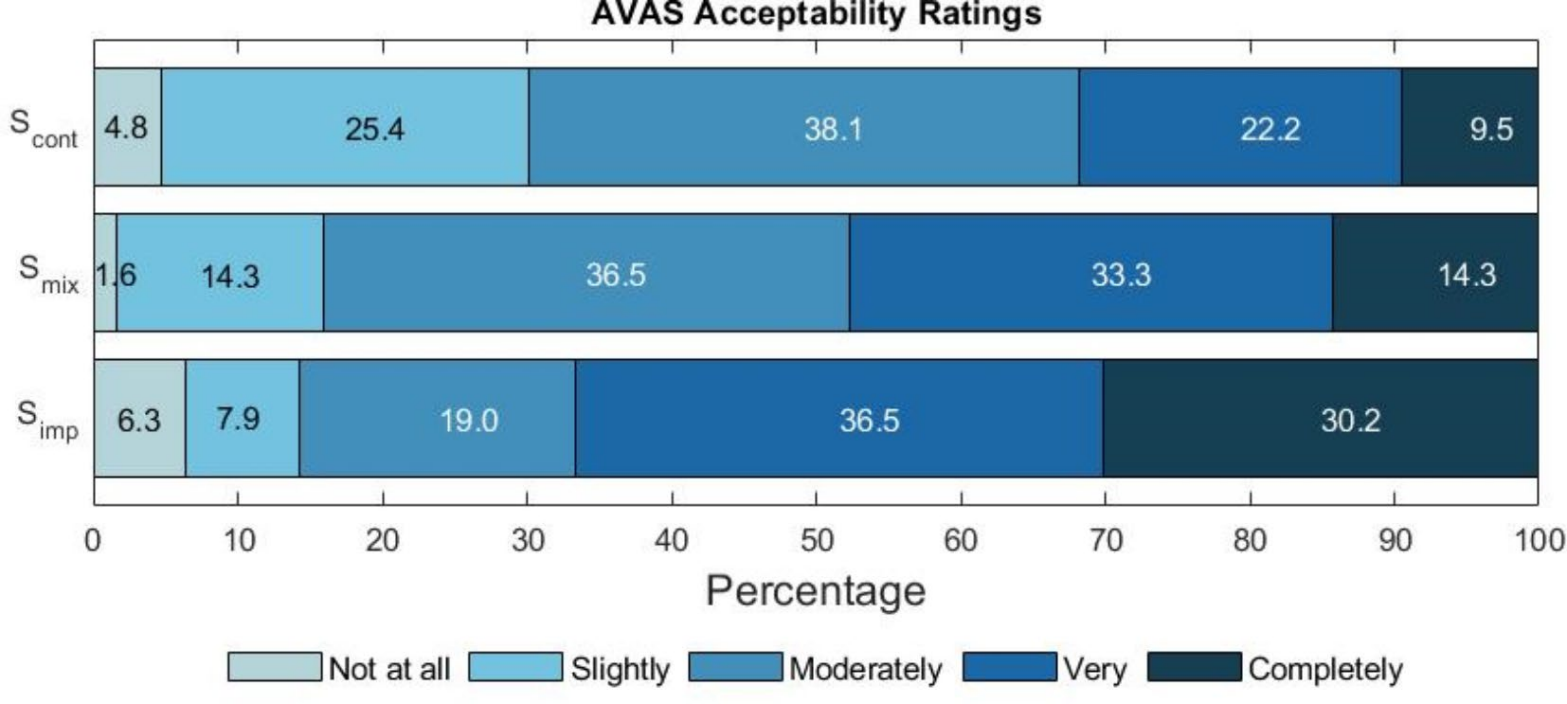
AVAS acceptability ratings in response to the question “Based on your experiences in VR, please rate each sound in terms of how acceptable it is for use as an e-scooter alert sound”.

An overview of the acceptability ratings is given in Fig. 7.

Non-parametric Wilcoxon signed-rank tests are used to quantify the significance of the acceptability differences between the stimuli. A significant difference is seen between S_imp_ and S_cont_ (*Z* = − 3.517, $p < 0.001$) and between S_mix_ and S_cont_ (*Z* = − 2.462, $p < 0.014$), but a non-significant difference is observed between S_mix_ and S_imp_ (*Z* = − 1.420, $p < 0.156$). Mann-Whitney U tests were conducted to determine whether there is a significant difference in acceptability ratings between the sighted and blind groups. The results indicate significant differences between these groups for S_cont_ (*U* = 342.50, *p* = 0.028) and S_imp_ (*U* = 271.50, *p* = 0.001), but a non-significant difference for S_mix_ (*U* = 402.00, *p* = 0.175).

The results presented in Fig. 7 show that for all three AVAS conditions, 70% or more of responses fall into the categories of ‘moderately’ to ‘completely’ acceptable, with 4% of total responses falling into the category of ‘not at all’ acceptable. S_imp_ shows the highest acceptability responses, with 86% of responses falling into the ‘moderately’ to ‘completely’ acceptable range, followed by S_mix_ (84%) and S_cont_ (70%). It is seen that S_imp_ has the highest proportion of both ‘completely’ acceptable (30%) and ‘not at all’ acceptable responses (6%), suggesting that this condition divides opinion more than the other sounds. When comparing acceptability ratings across visual acuity groups, the same ranks of conditions are seen, however the blind group favoured S_imp_ more strongly than the sighted group, with 52% of completely acceptable ratings for the blind group compared to 9% for the sighted group. Please refer to the supplementary information for more information regarding these differences.

#### Qualitative analysis

Analysis of the comment data was conducted using a thematic analysis approach^44^. All comments were first translated to English to enable analysis by the research team. The comments were then read and re-read by the research team, codes were identified (words or phrases that relate to a single aspect), before the codes were organised into recurrent themes. The following provides a high-level overview of the qualitative data, however, more information can be found within the supplementary information provided. For the participants who identified S_imp_ as their most preferred sound, a prominent theme in the responses was that this sound was the easiest to detect: “...[$S_{imp}$] *is easier to hear and I think it’s easier to hear in each environment”* [p44]. A related theme was that this sound was perceived as distinct and distinguishable: *“It’s different from all the city sounds, distinctive from normal traffic sounds...”* [p30]. Whilst preference for S_imp_ was typically associated with ease of detection due to its more distinct characteristics, preference for S_cont_ was typically associated with reduced annoyance in comparison to the other stimuli: “Even though [$S_{imp}$] *seems to be more easily identifiable, I prefer* [$S_{cont}$] *as it’s less annoying altogether”* [p23]. For preference of AVAS sound S_mix_, associated themes were distributed across both ease of detection and lack of annoyance:*“*I felt like it hit the perfect balance of recognizability without being too annoying...” [p49].

## Methods—field trials

To enhance results from the VR experiment presented above, field trials were conducted to evaluate pedestrian and rider perceptions of the e-scooter AVAS sounds in real-world use cases. The AVAS implementation consisted of a Hall effect system to gather speed information from the front wheel of the e-scooter, a Raspberry Pi running a Python script to modify playback of a WAV file with respect to speed, and a 30mm diameter Bluetooth loudspeaker attached to the stem. A calibration level of 62 $L_{\textrm{AFmax}}$ (2 m distance) was used, as based upon a 10 m detection distance in a 55 dB $L_{\textrm{Aeq}}$ environment^29^. More details of the setup can be found within the supplementary information, Section S5.

During the rider trials, participants were required to ride the e-scooter along a predetermined route of approximately 1.1 km (5 min), followed by a short questionnaire investigating their experiences. This was repeated for each of the three candidate AVAS sounds, with order of presentation balanced across participants. Following the third ride, an extra set of questions was presented. The rider trials were conducted within the University of Salford’s Peel Park Campus with a total of 11 individuals participating.

During the pedestrian trials, participants were required to walk from point A to point B, covering a distance of 75 m (110 s). During their journey, a member of the research team passed the pedestrian on an e-scooter from behind. The pass was at a speed of approximately 20 km/h and had a closest approach of approximately 2 m. Participants walked back to point A and another pass occurred. This was repeated for each of the three candidate AVAS sounds and a baseline condition (no AVAS), with order of presentation balanced across participants. Following each sound, the participants were required to complete a questionnaire detailing their experience of the e-scooter pass. Following the final pass, an extra set of questions was presented. The pedestrian trials were conducted within the University of Salford’s Peel Park Campus, with a corresponding noise level of 57 dB $L_{\textrm{Aeq}}$ over a representative 15 min measurement period. A total of 14 individuals participated in the pedestrian trials, including 4 participants who identified as registered blind or partially sighted.

## Results—field trials

Summarising the results, pedestrian responses on the topic of detectability revealed that the baseline condition (no AVAS) was insufficiently detectable, with 71.4% of participants disagreeing or strongly disagreeing that the baseline sound was sufficiently detectable for their needs. A total of 85.8% of participants agreed or strongly agreed that they were clearly alerted to an approaching e-scooter for the impulsive sound S_imp_, with the figure at 71.7% for the mixed impulsive and continuous sound S_mix_. When evaluating overall experience, 71.4% of pedestrians felt that the impulsive sound S_imp_ met their needs as a pedestrian, followed by 64.3% for S_mix_, 50.0% for S_cont_ and 21.4% for the baseline (no AVAS) condition.

When evaluating overall rider experience, the AVAS sounds were generally perceived as positive, with 81.8% of participants saying that sound S_mix_ positively influenced, or strongly positively influenced the overall riding experience. Riders predominantly felt that the AVAS sounds varied appropriately with the speed of the e-scooter, suggesting that the AVAS playback rate change of 1% plus specified level change was appropriate.

The results show that an e-scooter AVAS sound can be positive for both pedestrians and riders. Impulsive components are seen to improve perceived detectability for pedestrians, however riders also value continuous sound elements as these can lead to a greater rider experience in comparison to impulsive elements alone. As such, a mixed sound which contains both impulsive and continuous elements may be most suited for an optimised e-scooter AVAS sound. Please refer to the supplementary information, Section S6, for full results.

## Discussion

In this study, an immersive virtual reality experiment was developed to investigate perception of e-scooter AVAS sounds, with regards to detectability and annoyance. $N =$ 63 participants from 4 European countries completed the experiment, including 31 blind and partially sighted individuals. The experiment involved a series of tasks based on typical e-scooter scenarios, including detection of a pass from behind, detection of speed changes, detection in the presence of multiple e-scooters, and an acceptability task.

The results from the first detection task showed that missed detection rates were high for the baseline condition, i.e. e-scooters without an AVAS, with missed detection rates between 90 and 97% for all environments tested. This result indicates that additional alerting sounds are likely needed for auditory detection of e-scooters in typical city environments. With regards to AVAS level, the 56 dBA stimuli did not provide sufficient auditory warning, as based on the defined risk threshold of 10 m, with the exception of S_imp_ within the 49 dBA environment. The 66 dBA stimuli offered significant detectability improvement, resulting in significantly lower missed detections and sufficient detection distances within the 49 dBA and 55 dBA environments. These results suggest that if auditory detection at a safe distance is desired, the minimum requirements specified in UNECE Regulation 138 for quiet running vehicles^19^ may not be adequate, as also presented in^29^. When comparing AVAS characteristics with respect to detectability, S_mix_ resulted in a slightly reduced detection distance in comparison to the other sounds. This sound was characterised by a continuous ‘chord’, with impulsive components layered on top, at a rate of 1.15 Hz. As there were no significant differences in detectability between a purely continuous sound, and a purely impulsive sound, the reduced performance associated with S_mix_ is likely due to the slower impulse rate of the impulsive component of this sound in comparison to S_imp_, resulting in greater distances between the detectable impulses. Further work is needed to quantify the relationship between modulation rate and detectability for AVAS sounds, in order to extend current understanding^11,22^.

For the deceleration task, a playback rate increase of 2% per km/h may offer a small reduction in reaction time of speed change in comparison to the 1% condition, however, this difference appears to be small. For reaction times relating to detection of the e-scooter becoming stationary, a large significant difference was seen between AVAS modifications with level change compared to those without. Due to the large reaction times seen for modifiers without level changes (2–3 s), it is likely that participants were expecting the AVAS to stop when the e-scooter became stationary, and in the case where this did not happen, there was hesitation on detecting a stop. It is possible that training effects could reduce the differences seen. From these results, it is therefore recommended that AVAS systems reflect operational speed by means of both frequency and level modifications. If a stationary sound is desirable however, it is likely beneficial that this sound is distinct to the moving sound, as studied previously^35^.

For scenarios with multiple e-scooters, the most significant variable on detection rate and distance was AVAS speed dependence of the additional e-scooters. For speed independent AVAS conditions, the AVAS from all e-scooters within the scene were identical, resulting in difficulties in distinguishing e-scooters that may pose a hazard to the pedestrian. In the case where AVAS sounds were speed dependent, detection performance of an approaching e-scooter within a multiple e-scooter scenario was significantly improved. This result confirms the importance of modifying the AVAS to reflect speed, as it not only enables detection of acceleration and deceleration, but it also enables significantly better detectability in multiple vehicle scenarios. Differences in AVAS sound features were not seen to be significant with regards to detection distance for this task.

Acceptability ratings showed that more participants rated the AVAS sound with strong impulsive characteristics, S_imp_, as the most acceptable AVAS sound, however, all three sounds were rated as ‘moderately’ to ‘completely’ acceptable by 70% or more of the sample. When evaluating the qualitative responses given, acceptability ratings were primarily based on ease of detection and this is possibly due to the context of the acceptability rating task, which followed the detection tasks. Participants felt that the AVAS sounds containing continuous elements, S_cont_ and S_mix_, were not as detectable, which led to lower acceptability ratings, however, this was not fully backed up by significant differences within the quantitative detection data, for which only S_mix_ showed a small significant reduction in detectability during the first task. For participants who also based their acceptability judgements on annoyance, S_cont_ and S_mix_ were typically rated more highly. The low number of ‘not at all acceptable’ responses suggests that participants may find all sounds acceptable if they are deemed to be appropriately detectable. Based on these results, one option to balance detectability and annoyance would be to have a variable mix ratio of continuous and impulsive elements, so that in higher risk situations, such as at high speeds or within shared-use spaces, the AVAS is more dominated by impulsive components which are perceived as more detectable.

As well as acceptability from a pedestrian standpoint, an important consideration for e-scooter AVAS development is acceptability from a user standpoint, due to the increased exposure to the AVAS in comparison to pedestrians. The field trials revealed that an e-scooter AVAS sound can be positive for riders, with 81.8% of participants saying that sound S_mix_ positively influenced, or strongly positively influenced the overall riding experience. Whilst an impulsive AVAS sound was preferred by pedestrians during the field trials, a mixed sound which contains both impulsive and continuous elements may be most suited when taking into account both riders and pedestrians.

To conclude, this study has presented insight and design recommendations for micromobility AVAS sounds, as based on simulations of e-scooter movements within a virtual environment and field trials, considering sighted and blind participants. Furthermore, a virtual reality method was outlined, which combines 360 degree audio-visual recordings with animated computer generated graphics of vehicle movements, as well as auralisations including acoustic directivity data. This method has proved a useful tool to develop an understanding into the perception of vehicle acoustics within real-world use cases and could be utilised in further studies in the field. It is hoped that the results presented in this paper will help inform transportation policy makers going forward and enable wider adoption of micromobility.

## Supplementary Information

Supplementary Information 1.

Supplementary Information 2.

Supplementary Information 3.

Supplementary Information 4.

## Data Availability

The datasets generated during and/or analysed during the current study are available from the corresponding author on reasonable request.
