# Supplementary material for: Evaluation of auditory alerting systems for safe electric scooter operations
Source: Sci Rep. 2025 Jan 27;15:3424. doi: 10.1038/s41598-024-80975-1 (PMC11772828; doi:10.1038/s41598-024-80975-1)

Evaluation of auditory alerting systems for safe electric scooter operations

Supplementary Information

Tim Walton[1], Antonio J. Torija[1], Richard J. Hughes[1] and Andy S. Elliott[1]

[1] Acoustics Research Centre, University of Salford, The Crescent, Manchester, M5 4WT, United Kingdom

Supplementary information contents

S1. Additional information on 360 degree audio-visual recording locations.

S2. Tables of variable combinations for the three tasks completed.

S3. Acoustic Vehicle Alerting System (AVAS) acceptability ratings by visual acuity group.

S4. Selected additional quotations associated with identified thematic analysis themes.

S5. Acoustic Vehicle Alerting System (AVAS) implementation for field trials.

S6. Field trial additional results.

Attached video material

The following video clips represent the view of the participant within each section of the experiment, and as such, contain typical head movements. Please note, video artifacts are due to screen recording process and were not present in the Head-Mounted Display (HMD).

SV1 – Scene from Task 1 - Detection

SV2 – Scene from Task 2 - Deceleration

SV3 - Scene from Task 3 - Multiple Source

S1. Additional information on 360 degree audio-visual recording locations

S1.1 - ENV1

Stimuli Ref: ENV1
Description: City park environment, characterised by distant road traffic noise.
Calibration Level: 49 dB L_{Aeq}
Coordinates: 53.492453, -2.272207

Figure S1.1 Satellite imagery of location ENV1. Exact recording location specified by red arrow. Source: Google Maps (2023). https://maps.app.goo.gl/Nr7eqFpaky12HJFSA [Accessed 2 May 2023]

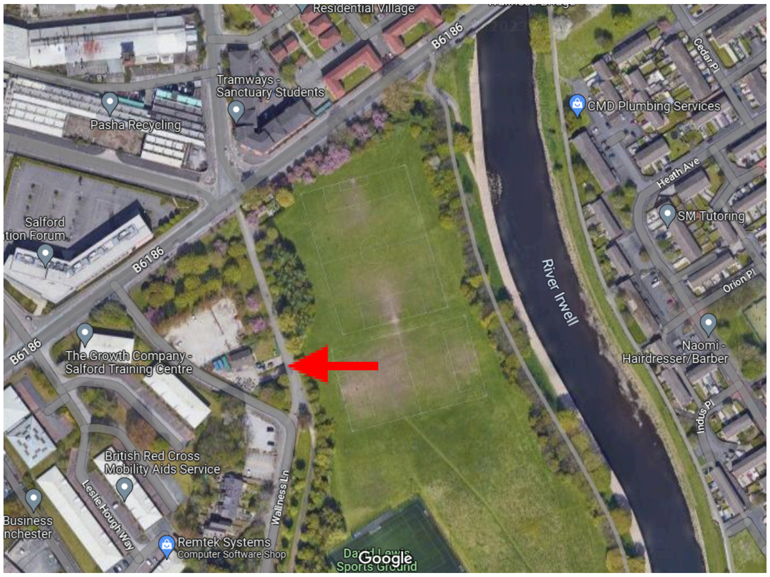

Figure S1.2 Screenshot of e-scooter graphic within ENV1 360 degree audio-visual recording.

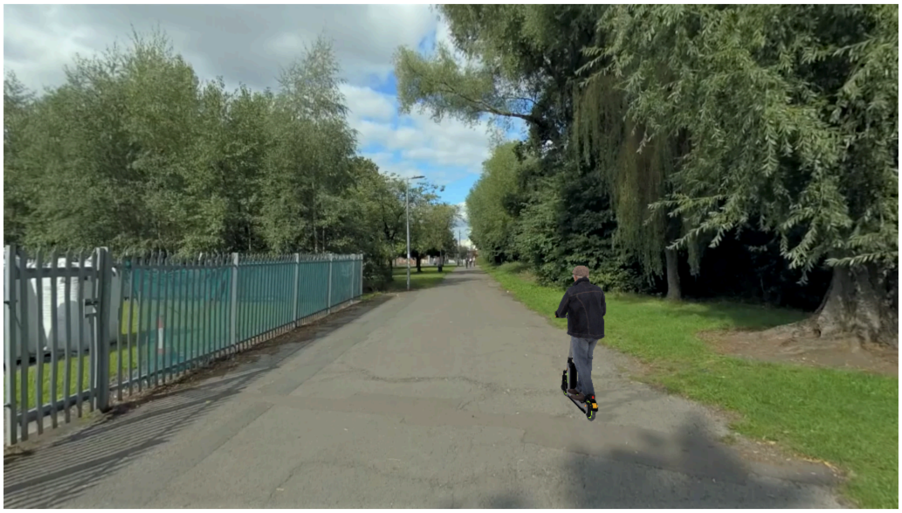

S1.2 – ENV2

Stimuli Ref: ENV2
Description: Shared use concourse, characterised by plant and machinery noise.
Calibration Level: 55 dB L_{Aeq}
Coordinates: 53.471938, -2.298394

Figure S1.3 Satellite imagery of location ENV2. Exact recording location specified by red arrow. Source: Google Maps (2023). https://maps.app.goo.gl/AnZANszSJcZTb5M58 [Accessed 2 May 2023]

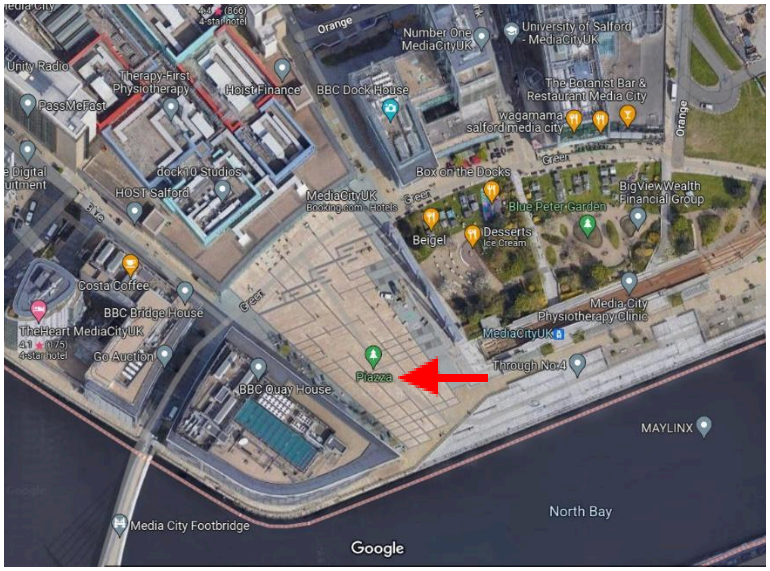

Figure S1.4 Screenshot of e-scooter graphics within ENV2 360 degree audio-visual recording.

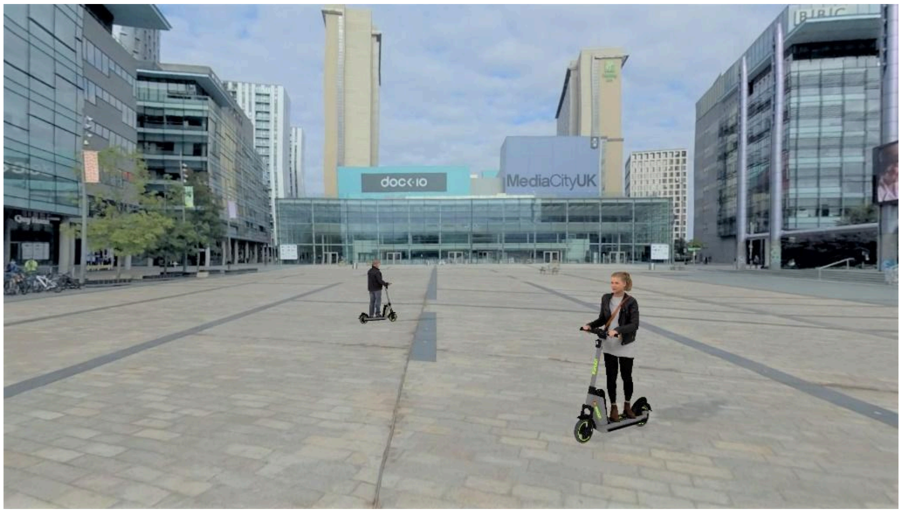

S1.3 – ENV3

Stimuli Ref: ENV3
Description: Busy city road, characterised by dominant road traffic noise.
Calibration Level: 70 dB L_{Aeq}
Coordinates: 53.484359, -2.271876

Figure S1.5 Satellite imagery of location ENV3. Exact recording location specified by red arrow. Source: Google Maps (2023). https://maps.app.goo.gl/fu1K26Q6LP8w9Bmz7 [Accessed 2 May 2023]

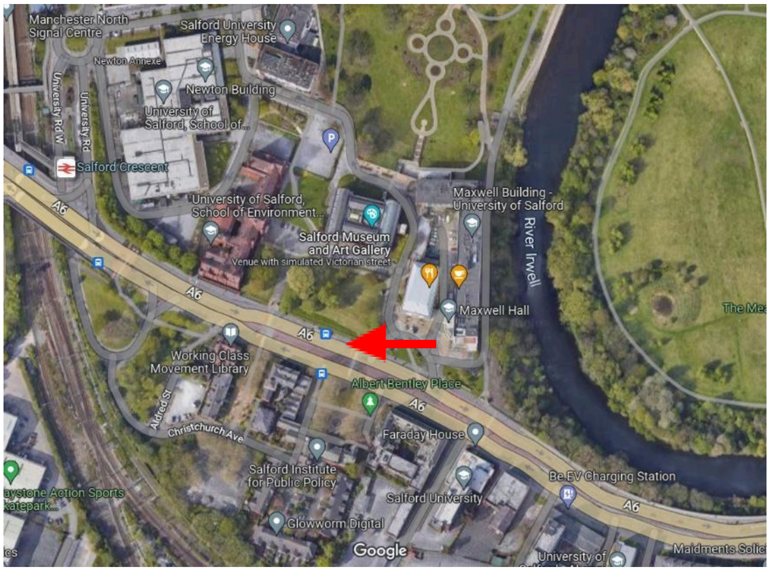

Figure S1.6 Screenshot of e-scooter graphic within ENV3 360 degree audio-visual recording.

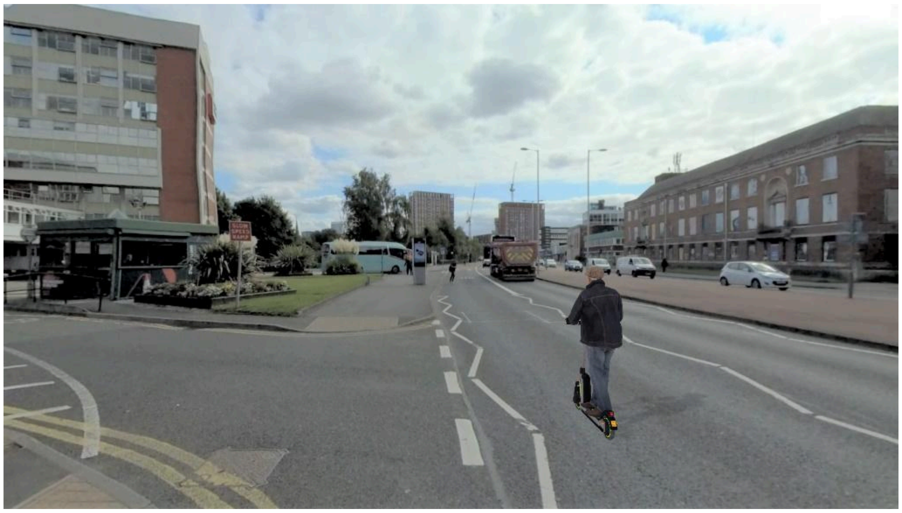

S2. Tables of variable combinations for the three tasks completed.

Table S2.1. Variable combinations for Task 1. Presentation of trials was randomised.

Combination	Environment	$AVAS_{type}$	$AVAS_{level}$ (dB)
1	ENV1	S_{base}	NA
2	ENV1	S_{cont}	56
3	ENV1	S_{cont}	66
4	ENV1	S_{imp}	56
5	ENV1	S_{imp}	66
6	ENV1	S_{mix}	56
7	ENV1	S_{mix}	66
8	ENV1	No Pass	No Pass
9	ENV1	No Pass	No Pass
10	ENV2	S_{base}	NA
11	ENV2	S_{cont}	56
12	ENV2	S_{cont}	66
13	ENV2	S_{imp}	56
14	ENV2	S_{imp}	66
15	ENV2	S_{mix}	56
16	ENV2	S_{mix}	66
17	ENV2	No Pass	No Pass
18	ENV2	No Pass	No Pass
19	ENV3	S_{base}	NA
20	ENV3	S_{cont}	56
21	ENV3	S_{cont}	66
22	ENV3	S_{imp}	56
23	ENV3	S_{imp}	66
24	ENV3	S_{mix}	56
25	ENV3	S_{mix}	66
26	ENV3	No Pass	No Pass
27	ENV3	No Pass	No Pass

Table S2.2. Variable combinations for Task 2. Presentation of trials was randomised. $AVAS_{mod}$ PR1 refers to a playback rate increase of 1% per km/h, PR1,L to a playback rate increase of 1% per km/h plus level change, PR2 to a playback rate increase of 2% per km/h and PR2,L to a playback rate increase of 2% per km/h plus level change.

**Combination**	**Environment**	**$AVAS_{type}$**	**$AVAS_{level}$ (dB)**	**$AVAS_{mod}$**
1	ENV1	S_{cont}	66	PR1
2	ENV1	S_{cont}	66	PR1,L
3	ENV1	S_{cont}	66	PR2
4	ENV1	S_{cont}	66	PR2,L
5	ENV1	S_{cont}	66	No Deceleration
6	ENV1	S_{imp}	66	PR1
7	ENV1	S_{imp}	66	PR1,L
8	ENV1	S_{imp}	66	PR2
9	ENV1	S_{imp}	66	PR2,L
10	ENV1	S_{imp}	66	No Deceleration
11	ENV1	S_{mix}	66	PR1
12	ENV1	S_{mix}	66	PR1,L
13	ENV1	S_{mix}	66	PR2
14	ENV1	S_{mix}	66	PR2,L
15	ENV1	S_{mix}	66	No Deceleration

Table S2.3. Variable combinations for Task 3. Presentation of trials was randomised. For speed independent conditions, all e-scooters within the scene had an AVAS which represented 20 km/h. For speed dependent conditions, the frontal e-scooters had an AVAS with playback rate increase of 1% per km/h to reflect a speed of 13 km/h.

**Combination**	**Environment**	**$AVAS_{type}$**	**$AVAS_{level}$ (dB)**	**$AVAS_{speed}$**
1	ENV2	S_{cont}	66	Speed independent
2	ENV2	S_{cont}	66	Speed dependent
3	ENV2	S_{cont}	66	No Pass
4	ENV2	S_{cont}	66	No Pass
5	ENV2	S_{imp}	66	Speed independent
6	ENV2	S_{imp}	66	Speed dependent
7	ENV2	S_{imp}	66	No Pass
8	ENV2	S_{imp}	66	No Pass
9	ENV2	S_{mix}	66	Speed independent
10	ENV2	S_{mix}	66	Speed dependent
11	ENV2	S_{mix}	66	No Pass
12	ENV2	S_{mix}	66	No Pass

S3. AVAS acceptability ratings by visual acuity group.

Figure S3.1 AVAS acceptability ratings for sighted participants in response to the question "Based on your experiences in VR, please rate each sound in terms of how acceptable it is for use as an e-scooter alert sound".

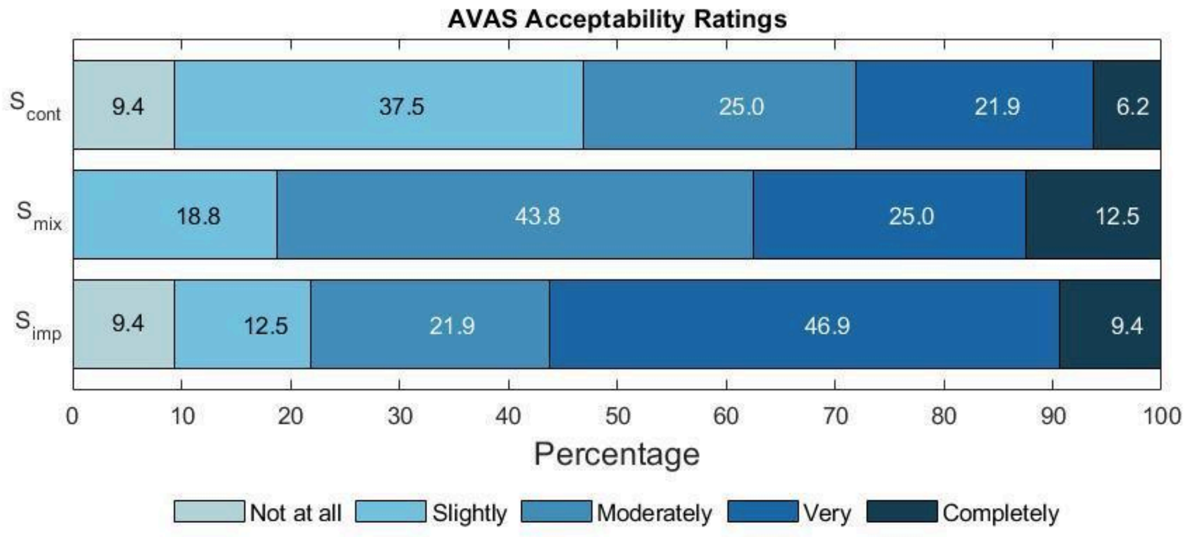

Figure S3.2 AVAS acceptability ratings for blind and partially sighted participants in response to the question "Based on your experiences in VR, please rate each sound in terms of how acceptable it is for use as an e-scooter alert sound".

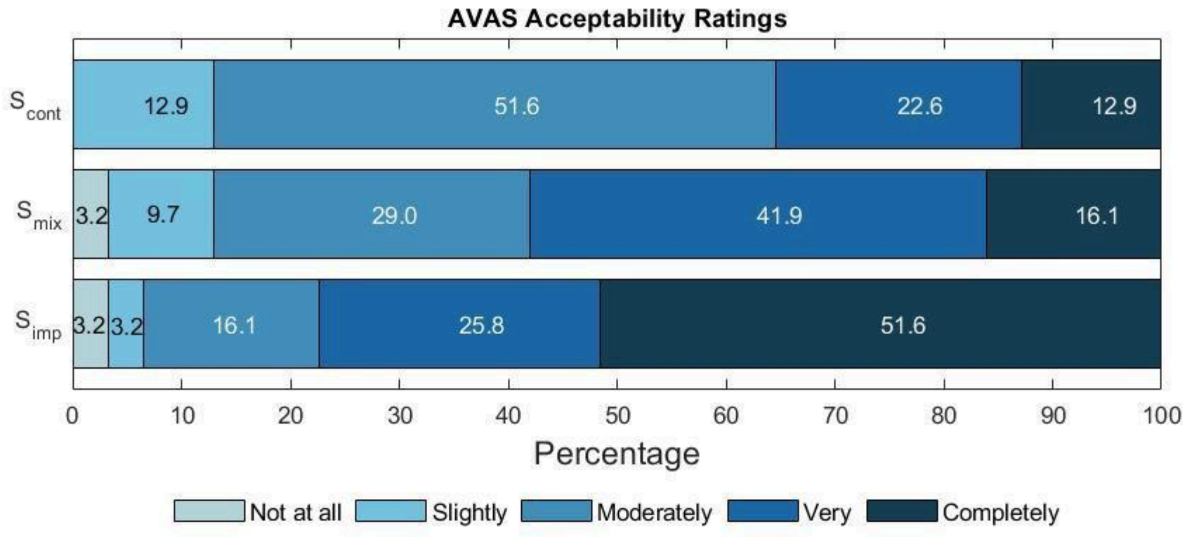

S4. Selected additional quotations associated with identified thematic analysis themes.

Table S4.1 Selected additional quotations associated with identified thematic analysis themes, for preferred AVAS S_{imp}. Typographical errors have been corrected for legibility.

ID	Preferred AVAS	Theme	Quotation
8	S_{imp}	Distinct	*"…clearer and more distinguished…I would instantly recognise it"*
10	S_{imp}	Distinct	*"Easier to distinguish from the background noise."*
11	S_{imp}	Distinct	*"…clearly distinguishable in most real-life scenarios."*
14	S_{imp}	Distinct	*"…it was the easiest to distinguish…"*
30	S_{imp}	Distinct	*"It's different from all the city sounds distinctive from normal traffic sounds."*
52	S_{imp}	Distinct	*"…differs more from other noises in the environment…"*
47	S_{imp}	Distinct	*"It was the most distinguishable"*
2	S_{imp}	Alerting	*"…gives more warning to a scooter behind you."*
7	S_{imp}	Alerting	*"…alerting the resident compared to others."*
12	S_{imp}	Alerting	*"…sounds like alarm and makes people alert."*
17	S_{imp}	Alerting	*"…it can be easily noticed from very behind."*
24	S_{imp}	Alerting	*"…easily recognisable that something like a hazard getting closer to you."*
26	S_{imp}	Alerting	*"…help to seek attention."*
2	S_{imp}	Rhythm	*"I prefer [S_{imp}] because it is pulsating a lot more…"*
9	S_{imp}	Rhythm	*"…the rhythm is more suitable…"*
18	S_{imp}	Rhythm	*"…you can work out the location of the oncoming scooter by listening to changes in the number of beeps."*
21	S_{imp}	Rhythm	*"Good pulse or beat"*
30	S_{imp}	Rhythm	*"…I like the way it pulses."*
31	S_{imp}	Rhythm	*"…I think it is easier to detect as it has a pattern or rhythm."*
43	S_{imp}	Stressful	*"The only downside with it is that it could be stressful"*
44	S_{imp}	Stressful	*"The only bad thing is that it may create some stress because it's irritating."*

Table S4.2 Selected additional quotations associated with identified thematic analysis themes, for preferred AVAS S_{cont}. Typographical errors have been corrected for legibility.

ID	Preferred AVAS	Theme	Quotation
23	S_{cont}	Annoyance	*"I prefer [S_{cont}] as it's a little less annoying altogether."*
29	S_{cont}	Annoyance	*"… it is less clangy and might be more acceptable to the driver of the scooter"*
41	S_{cont}	Annoyance	*"[S_{imp}] was very annoying especially with multiple scooters. [S_{mix}] I also found relatively annoying if I had to listen to it for 10min when on a scooter…"*
56	S_{cont}	Annoyance / Alerting	*"It is heard well and from afar without disturbing."*
13	S_{cont}	Annoyance / Alerting	*"[S_{cont}] would be my preference as its a little less siren based and still easy to pick up on."*

Table S4.3 Selected additional quotations associated with identified thematic analysis themes, for preferred AVAS S_{mix}. Typographical errors have been corrected for legibility. *ID is greater than total number of participants as a subset of participants had IDs assigned, but did not complete the experiment.

ID	Preferred AVAS	Theme	Quotation
16	S_{mix}	Detectability	*"Since there is variation in the level and tone I feel like [S_{mix}] is more easy to identify compared to other sounds."*
28	S_{mix}	Detectability	*"…it seemed to be the easiest for me to pick up from a distance."*
62	S_{mix}	Detectability	*"…I think it's easier to listen further away."*
64*	S_{mix}	Detectability	*"…best heard in different conditions."*
49	S_{mix}	Detectability / Annoyance	*"I felt like it hit the perfect balance of recognizability without being too annoying."*
6	S_{mix}	Detectability / Annoyance	*"…most identifiable from a distance… [S_{imp}] is the most annoying."*
5	S_{mix}	Annoyance	*"…I think this sound is not at all annoying and it sounds normal."*
22	S_{mix}	Annoyance	*"I rated these sounds imagining how annoying they would be and just in general how much I liked or disliked the sound."*

S5. Acoustic Vehicle Alerting System (AVAS) implementation for field trials.

Note S5.1 Description of AVAS implementation for field trials.

The schematic diagram presented in Figure S5.2 gives an overview of the hardware implementation used, with photographs of the setup seen in Figure S5.3. The system was built around a Raspberry Pi 4 Model B and a Hall effect system to gather speed information from the front wheel of the e-scooter. Two magnets on opposing spokes were used to allow sufficient speed sampling rates at low speeds, in combination with a KY-003 magnetic Hall effect switch, which was positioned on the front fork. The Hall effect switch was connected to the Raspberry Pi via the GPIO pins, with the Raspberry Pi being placed in a weatherproof case and attached to the lower stem of the e-scooter. The Raspberry Pi was connected wirelessly to a small, portable Bluetooth loudspeaker (30mm diameter, 250mAh, 1W, brand: Mohomo), which was attached to the stem of the e-scooter in a forward facing direction. The specifications of this loudspeaker driver can be considered as representative to the native driver onboard the e-scooter.

The processing was achieved via a Python script, which takes an audio WAV file as an input, along with RPM information from the Hall effect sensor, and modifies the audio file according to the set parameters. To play and modify the audio, the media player 'MPV' is used. A playback rate change of 1% per km/h was used, as this produced a smoother sounding pitch shift with the speed sensing setup used, in comparison to 2% per km/h). A speed dependent level algorithm was also used, with the AVAS being disabled below 5 km/h and at maximum volume at 20 km/h. This allowed a smooth AVAS onset when pushing off with the e-scooter and no additional alert sound when walking with the e-scooter. The level dependence with respect to speed is presented in Figure S5.4.

Figure S5.2 Schematic diagram showing components of AVAS implementation.

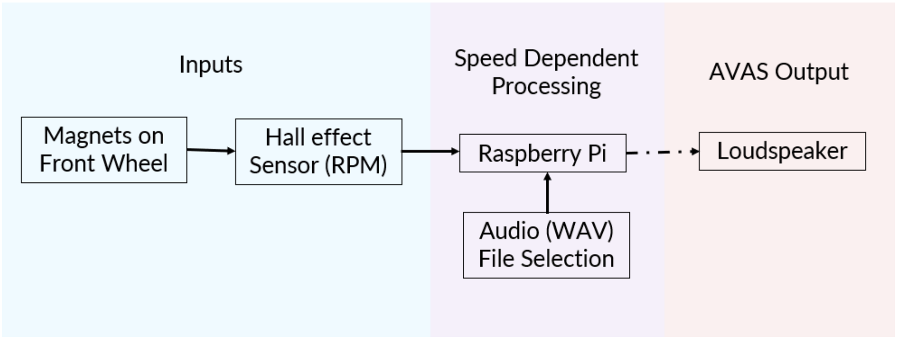

Figure S5.3 Photographs showing AVAS implementation on e-scooter. Note: Photographs have been edited to remove logos. 1-2) Magnets for speed sensor; 3) Hall effect sensor; 4) Raspberry Pi; 5) Portable battery pack for Raspberry Pi; 6) Bluetooth loudspeaker and additional battery via USB.

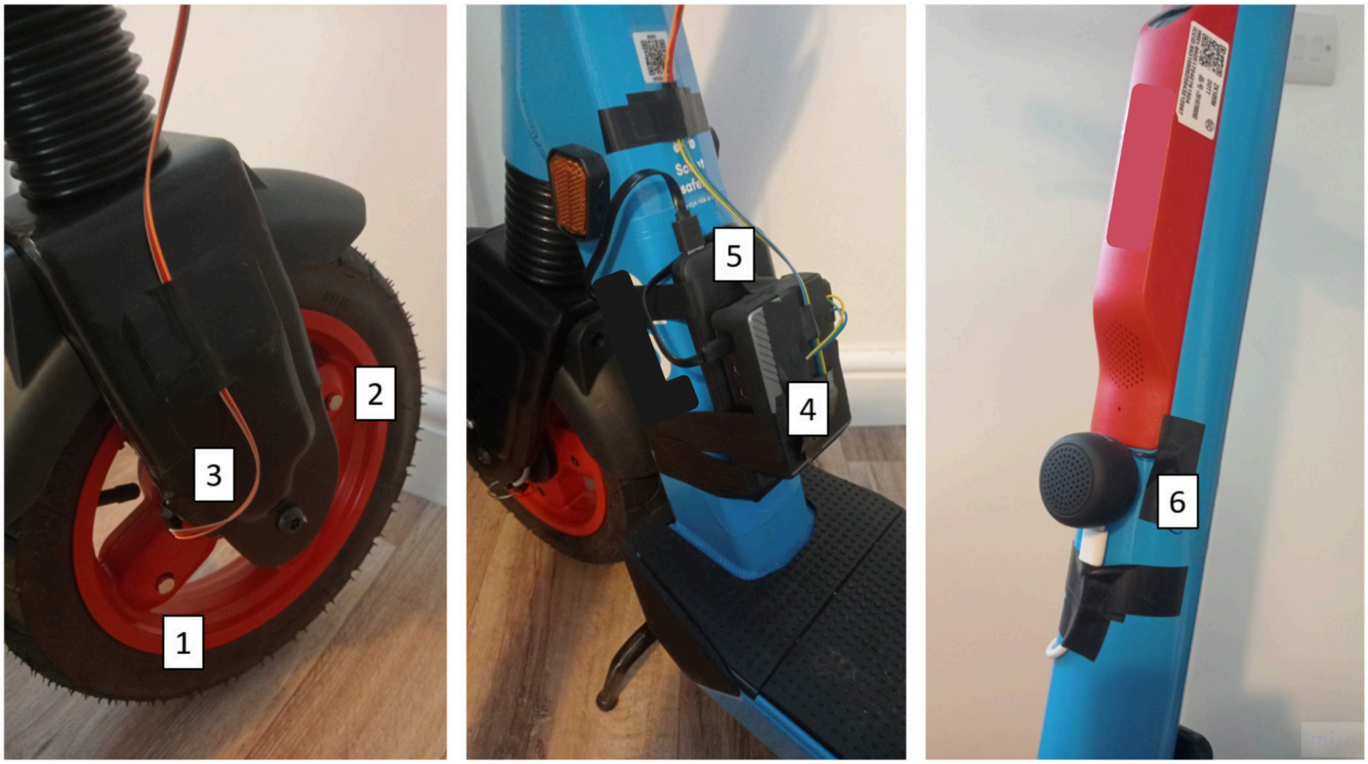

Figure S5.4 Relative AVAS gain with respect to speed for AVAS implementation. Note, AVAS commences from 5 km/h and reaches maximum volume at 20 km/h.

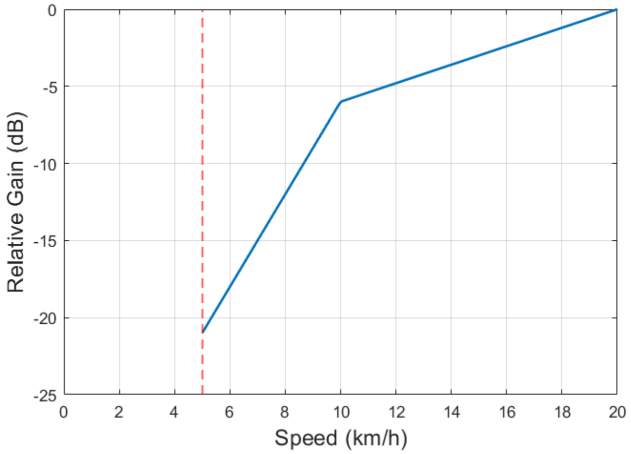

S6. Field trial additional results.

Figure S6.1 Questionnaire responses relating to detectability. For pedestrian responses, N=14, for rider responses, N=11.

a)

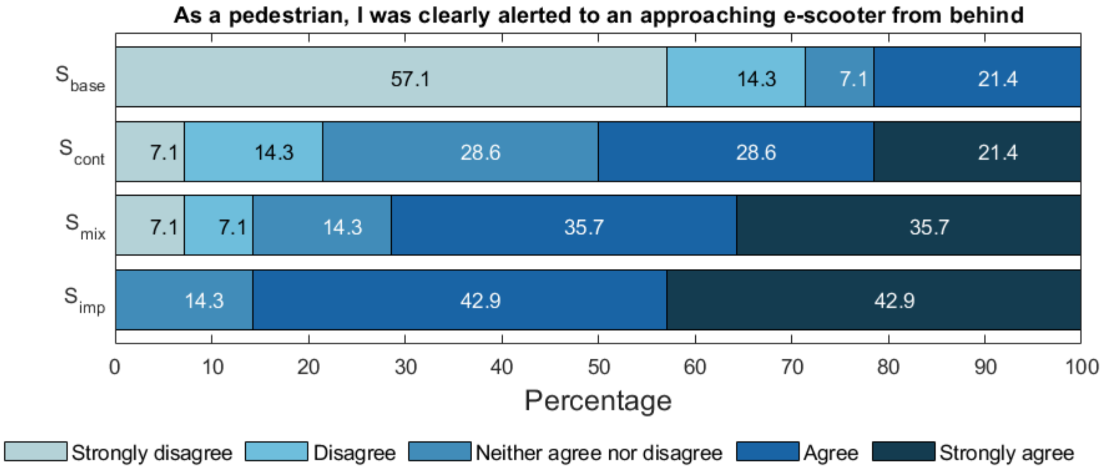

b)

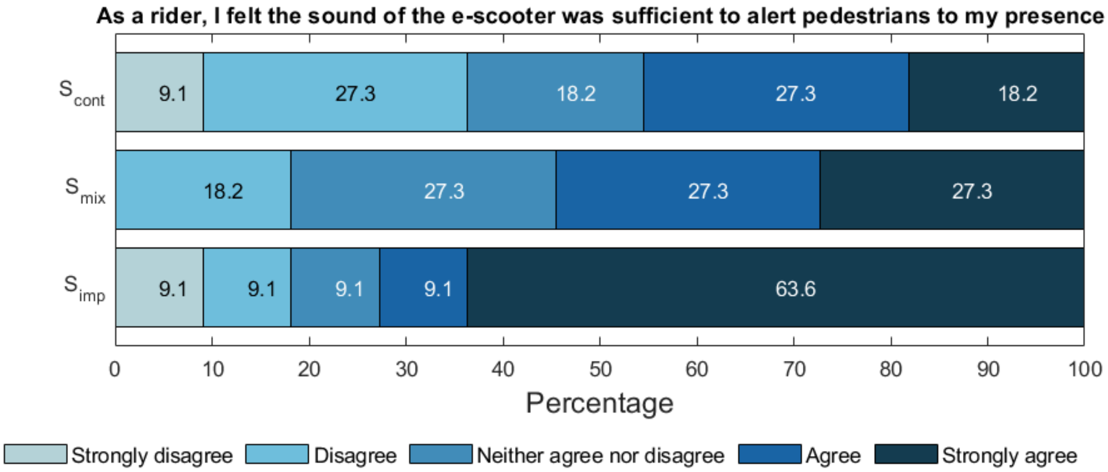

Figure S6.2 Questionnaire responses relating to loudness. For pedestrian responses, N=14, for rider responses, N=11.

a)

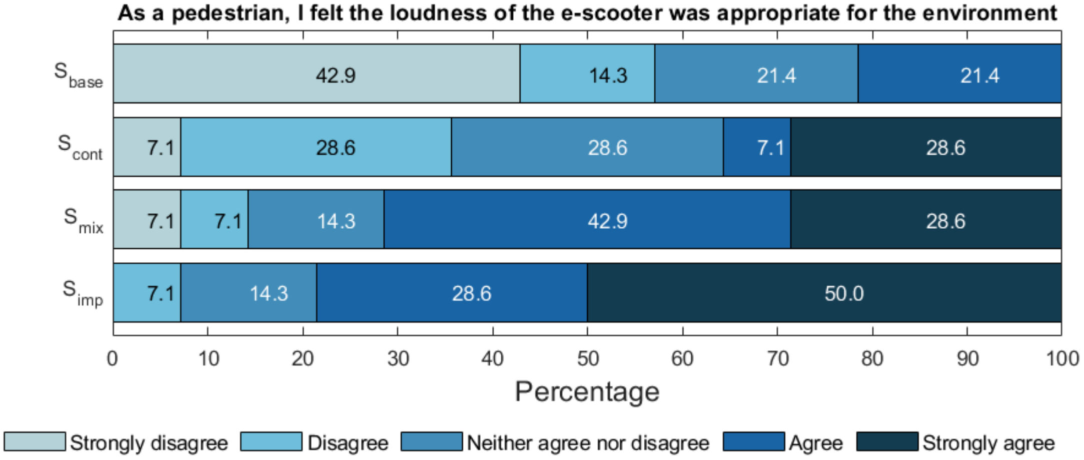

b)

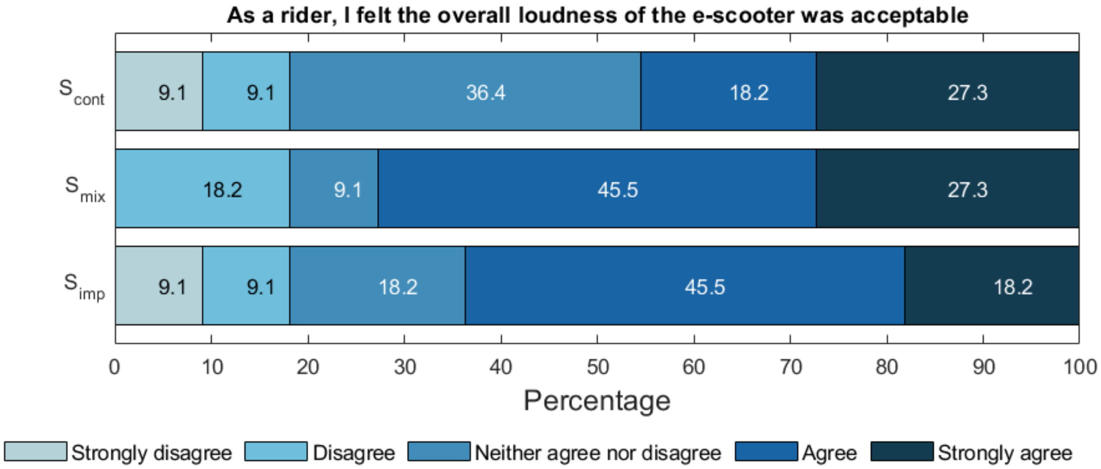

Figure S6.3 Questionnaire responses relating to sound character. For rider responses, N=11, for pedestrian responses, N=14.

a)

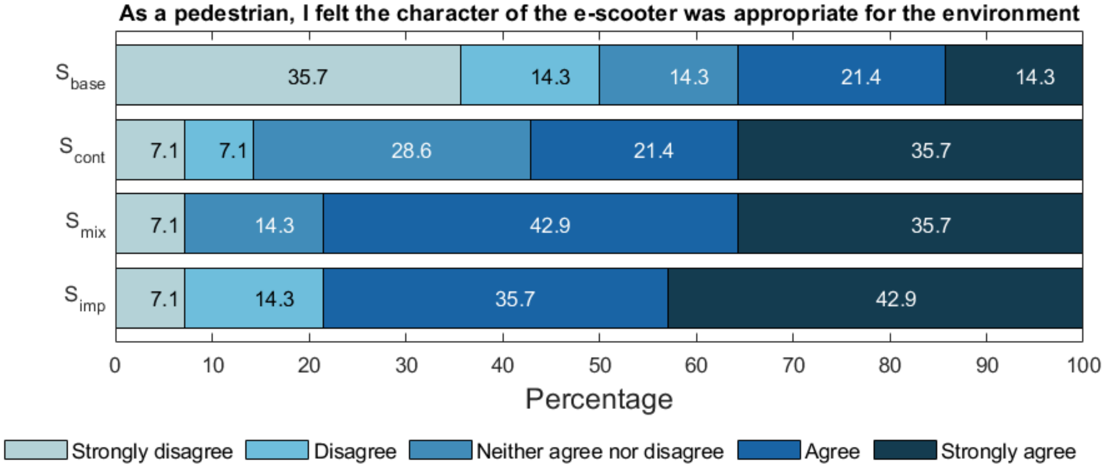

b)

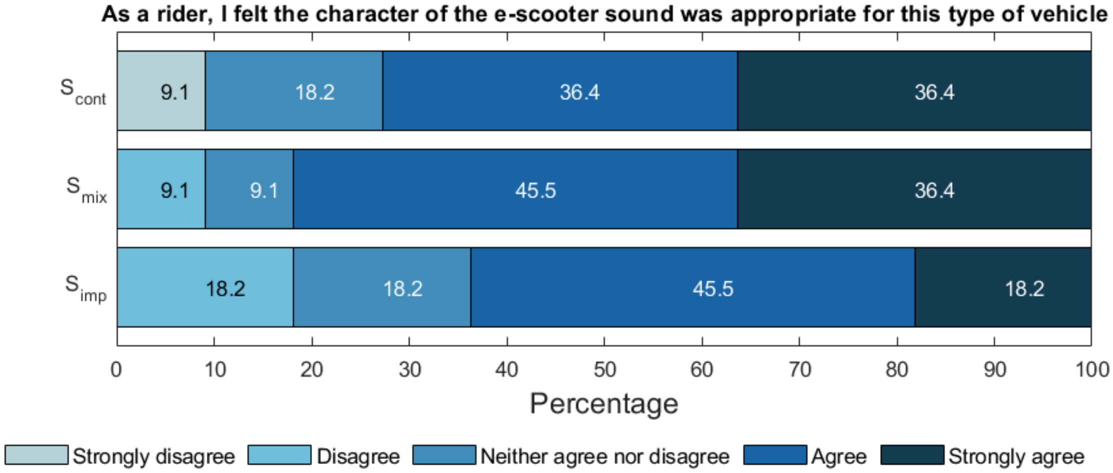

Figure S6.4 Questionnaire responses relating to rider auditory feedback. For rider responses, N=11.

a)

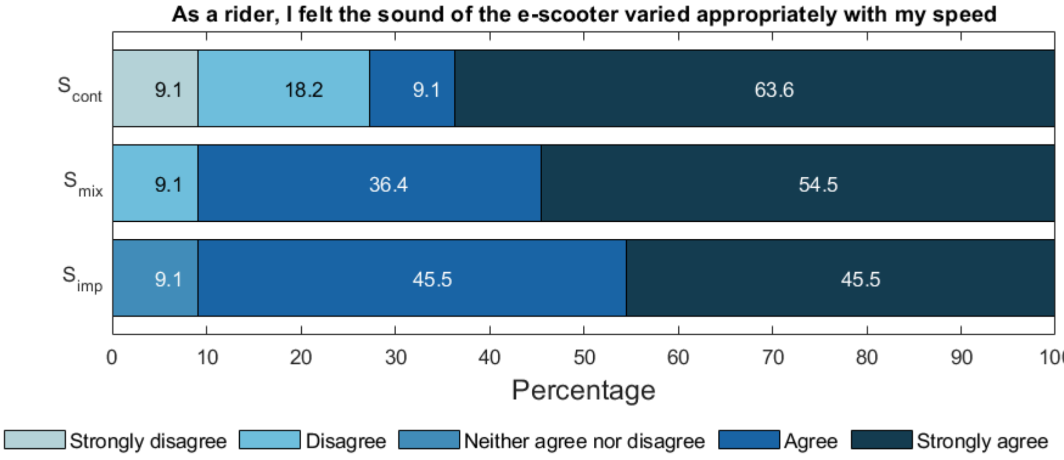

b)

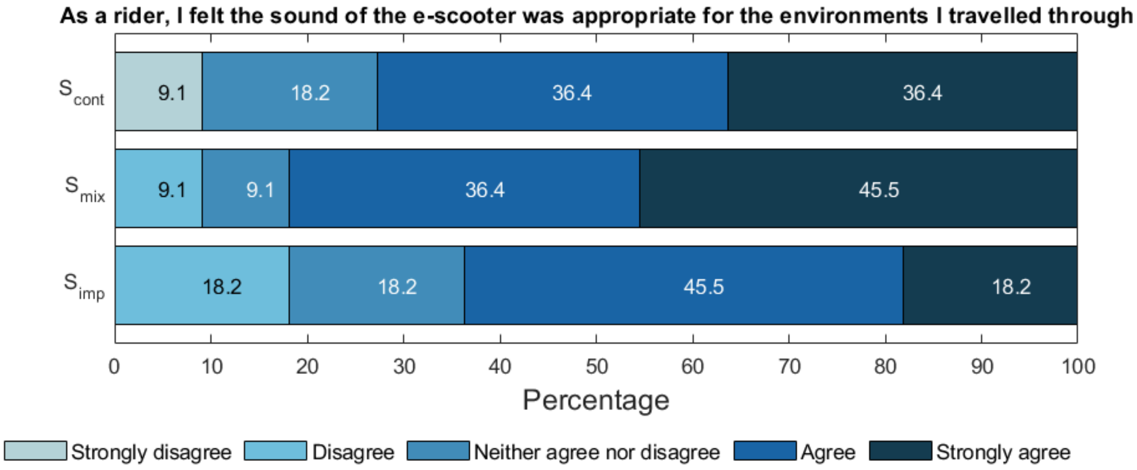

Figure S6.5 Questionnaire responses relating to overall experience. For rider responses, N=11, for pedestrian responses, N=14.

a)

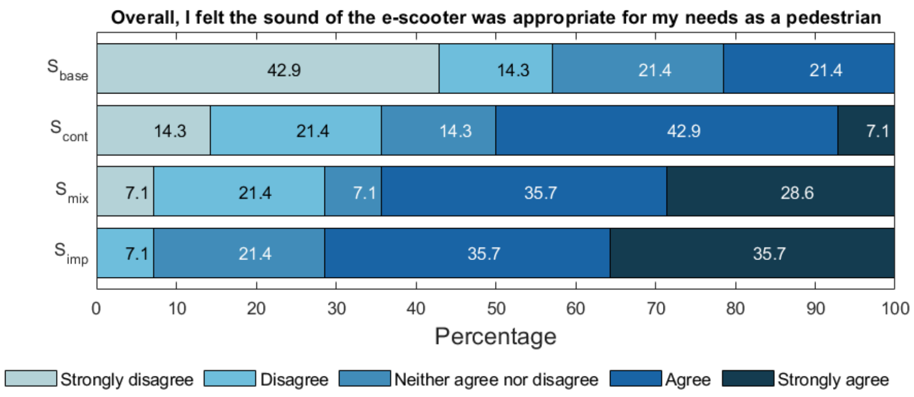

b)

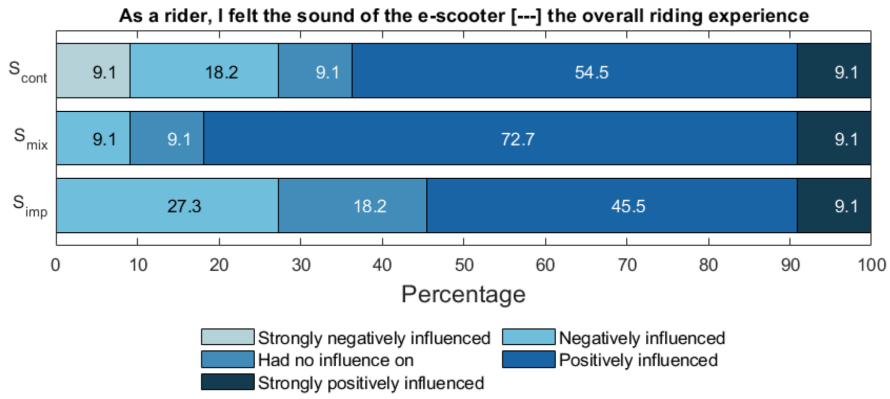

c)

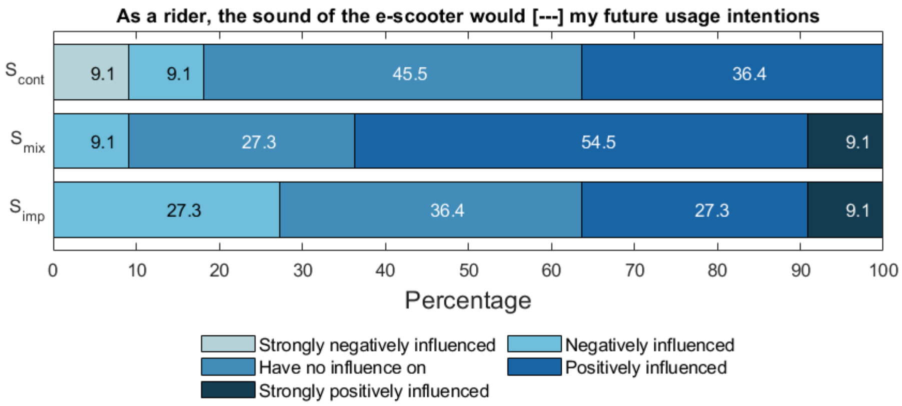

Figure S6.6 Questionnaire responses relating to overall preference for each AVAS sound tested. For rider responses, N=11, for pedestrian responses, N=14.

a)

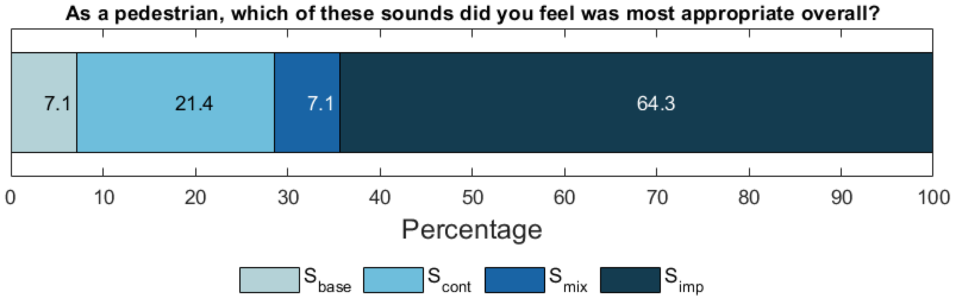

b)

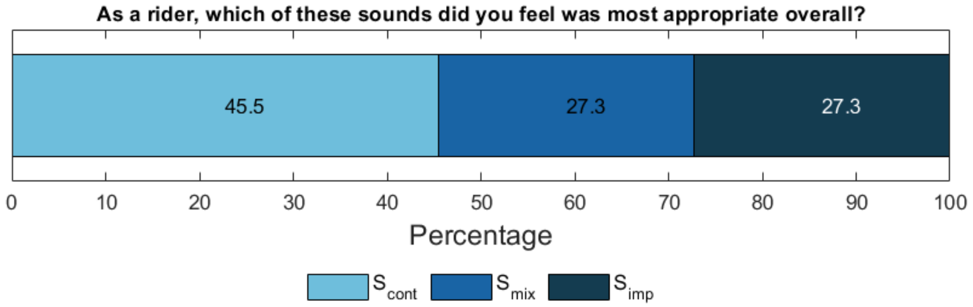

Supplement: Supplementary file 1 — Supplementary Information 1. [file 41598_2024_80975_MOESM1_ESM.pdf]
